# Biomimetic coating-free surfaces for long-term entrapment of air under wetting liquids

**DOI:** 10.1038/s41467-018-05895-x

**Published:** 2018-09-06

**Authors:** Eddy M. Domingues, Sankara Arunachalam, Jamilya Nauruzbayeva, Himanshu Mishra

**Affiliations:** King Abdullah University of Science and Technology (KAUST), Water Desalination and Reuse Center (WDRC), Biological and Environmental Science and Engineering (BESE) Division, Thuwal, 23955-6900 Saudi Arabia

## Abstract

Trapping air at the solid–liquid interface is a promising strategy for reducing frictional drag and desalting water, although it has thus far remained unachievable without perfluorinated coatings. Here, we report on biomimetic microtextures composed of doubly reentrant cavities (DRCs) and reentrant cavities (RCs) that can enable even intrinsically wetting materials to entrap air for long periods upon immersion in liquids. Using SiO_2_/Si wafers as the model system, we demonstrate that while the air entrapped in simple cylindrical cavities immersed in hexadecane is lost after 0.2 s, the air entrapped in the DRCs remained intact even after 27 days (~10^6^ s). To understand the factors and mechanisms underlying this ten-million-fold enhancement, we compared the behaviors of DRCs, RCs and simple cavities of circular and non-circular shapes on immersion in liquids of low and high vapor pressures through high-speed imaging, confocal microscopy, and pressure cells. Those results might advance the development of coating-free liquid repellent surfaces.

## Abstract (web summary)

Trapping air at the solid-liquid interface is a promising strategy for reducing frictional drag but could not be realized without perfluorinated coatings so-far. Here the authors demonstrate a biomimetic coating-free approach for entrapping air for long periods upon immersion in liquids.

## Introduction

Many industries, including oil and gas, desalination, irrigation, fracking, and wastewater treatment, routinely transport large quantities of liquids through pipes and suffer from reduced energy efficiency due to the frictional drag at the liquid-solid interface^1,2^. As a solution, polymeric additives have been used to facilitate slippage in pipes^1^. However, the use of such drag-reducing polymers is currently limited to high-value industries, such as oil and gas, because of their prohibitive costs, ~US $0.3 per m^3^ ^1^. As a consequence, numerous industries use pipes treated with perfluorinated coatings with non-specific textures to reduce drag under laminar and turbulent conditions^1,3–11^. The efficacy of such coatings is typically assessed by quantifying the contact angles of drops of water and a representative apolar liquid, such as hexadecane, in air. Based on the measurements of advancing angles, *θ*_A_, receding angles, *θ*_R_, and (advancing) static angles, *θ*_r_, the coatings are categorized as hydrophobic (if for water, *θ*_r_ > 90°^12^), superhydrophobic (if for water, *θ*_r_ > 150° and *θ*_A_ - *θ*_R_ < 5°^13^), omniphobic (if for both water and hexadecane, *θ*_r_ > 90°^14^), or superomniphobic (if for water and hexadecane, *θ*_r_ > 150° and *θ*_A_–*θ*_R_ < 5°^15^). Given that it has been experimentally demonstrated that the entrapment of air bubbles in microtextures on superhydrophobic and superomniphobic coatings decreases drag^3,4,8–10,16,17^ and boosts the efficiency of desalination processes^11,18–20^, these types of coatings are highly sought after in engineering applications.

Unfortunately, coating-based solutions are currently limited due to their high cost, non-biodegradability, and vulnerability to abrasive conditions or elevated temperatures^21–24^. For example, the productivity of superhydrophobic desalination membranes significantly degrades even under mild changes in temperature, for instance from 343 K to 363 K, due to the damage caused by the heat to the organosilane coating^22^. Alternative strategies to realize omniphobicity and superomniphobicity at solid-liquid interfaces that do not entirely depend on the chemical makeup of the coating would resolve these limitations and also improve the efficiency of the pipes.

Following the above-mentioned definitions of omniphobicity and superomniphobicity, it has been predicted and demonstrated that intrinsically wetting materials with microtextures composed of mushroom-shaped ‘reentrant’ pillars^25–31^ (Supplementary Figure 1A) and doubly reentrant pillars^15^ (Supplementary Figure 1B) could be rendered superomniphobic in air (*θ*_r_ > 150° and *θ*_A_–*θ*_R_ < 5° for both water and hexadecane). This earlier research heralded the possibility that the wetting properties of surfaces could be tailored through their microtextures with minimal dependence on surface chemistry. However, thermodynamic equilibrium mandates that the liquid will eventually penetrate into the microtexture when rough surfaces are made of intrinsically wetting materials^30,32^. It was therefore crucial for us to design microtextures that entrap air in metastable states, also known as the Cassie states^33^, for long periods by preventing wetting transitions^34^ leading to the fully filled or Wenzel state^35^.

**Fig. 1:**
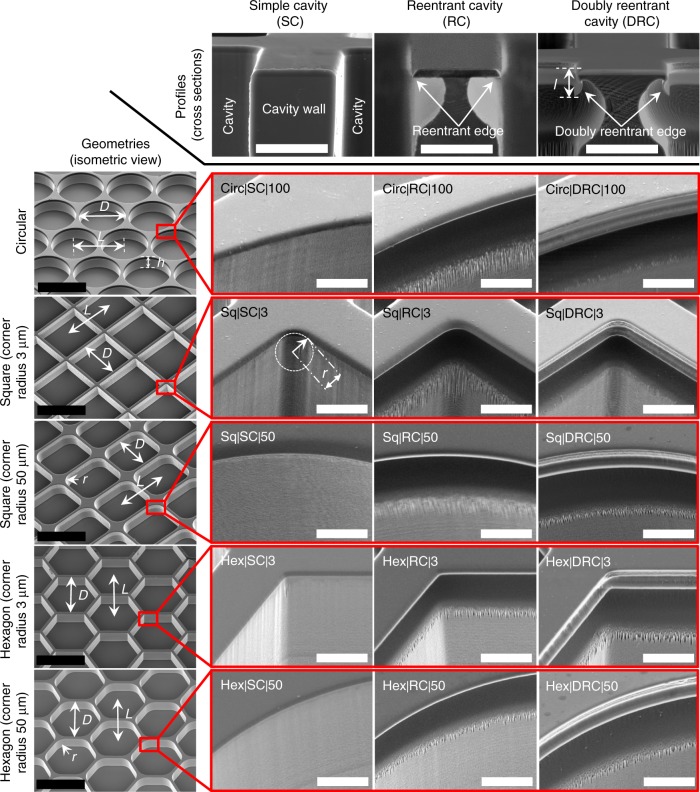
Scanning electron micrographs of fifteen types of microtextures investigated in this work. The cavities were classified based on their profiles as simple cavities (SCs), reentrant cavities (RCs), and doubly reentrant cavities (DRCs). Further, for each profile, circular, square, and hexagonal shapes were tested. The square and hexagonal shapes were further classified based on the sharpness of the corners. Scale bars: (i) Solid black line refers to 200 µm, and (ii) White line refers to 10 µm

To design such strategies, we sought inspiration from nature by analyzing the omniphobic cuticle of springtails (Collembola), soil-dwelling six-legged animals^36^. The hierarchical texture of the springtails’ cuticles, comprising bristles, papillose secondary granules, and nanoscopic comb patterns^37^, gives rise to structural colors (iridescence)^36^ and also enables plastron formation upon immersion in wetting liquids^38^. Following the alignment patterns on the springtail’s cuticle, we hybridized the shapes of doubly reentrant pillars with those on insects’ cuticles to create arrays of doubly reentrant cavities (DRCs) on intrinsically wetting silica surfaces (Fig. 1). Remarkably, we found that our DRCs demonstrated maximum robustness at entrapping air upon immersion in wetting liquids compared with any other known microtexture. Here, in a quest to understand the intricacies involved in trapping air and sustaining this entrapment, we present a detailed report on the mechanisms of our DRCs when immersed in wetting liquids. We compared intrinsically wetting surfaces adorned with cavities of different profiles (DRCs, RCs, and simple cavities (SCs)), shapes (circular, square, and hexagonal), and sharpness of corners.

## Results

### Classification of microtextures

We investigated arrays of cavities of varying profiles (DRCs, RCs, and SCs) and shapes (circular, square, and hexagonal) microfabricated onto silica surfaces (Fig. 1, Methods). To probe the effects of corners on the stability of trapped air, we further categorized square and hexagonal cavities by the sharpness of their corners, *r* = 3 μm and *r* = 50 μm. All the cavities investigated in this work had the primary dimension, *D* = 200 μm, defined as the diameter for circles, the edge length for squares, and the distance between apposing edges for hexagons, where depth was, *h* ≈ 50 μm (Table 1, Fig. 1 and Supplementary Figure 2). We chose the center-to-center distance between adjacent cavities, pitch, to be *L* = *D* + 12 μm. Those dimensions allowed us to minimize the real liquid-solid contact area, *A*_LS_ = *A*_H *×* _*ϕ*_LS_, and maximize the liquid-vapor contact area, *A*_LV_ = *A*_H *×* _*ϕ*_LV_, while maintaining a tight control over the microfabrication process, where *A*_H_ was the projected area of the surface, *ϕ*_LV_ and *ϕ*_LS_ were the area fractions of the liquid-vapor and liquid-solid interfaces (Supplementary Figure 3). We adopted the following nomenclature to refer to the cavities: “X|Y|Z”, where “X” is the shape (circular (Circ), square (Sq), or hexagonal (Hex)), “Y” is the type of the cavity or profile, DRC, RC, and SC, and “Z” is the corner radius in microns. For example Sq|DRC|50 refers to a surface with an array of square DRCs with a 50 μm corner radius. We chose hexadecane (intrinsic contact angle on silica in air, *θ*_o_ ≈ 20^°^) and water (*θ*_o_ ≈ 40^°^) as the representative liquids because of their ease of usage and differences in physical properties, especially vapor pressure and surface tension (Supplementary Table 1).

**Table 1:** A summary of all the experimental data presented in this work

Simple cavities (SCs)			Reentrant cavities (RCs)		Doubly reentrant cavities (DRCs)	
	Water	Hexadecane	Water	Hexadecane	Water	Hexadecane
*Circular (Circ|100)*						
*θ*_Pr_	130°	90°	130°	127°	128°	125°
*θ*_r_	113° ± 3°	65° ± 1°	125° ± 3°	118° ± 5°	124° ± 2°	114° ± 3°
*θ*_A_	118° ± 4°	72° ± 4°	141° ± 2°	137° ± 2°	139° ± 3°	134° ± 4°
*ϕ*_LV_	0.81	0	0.81	0.81	0.81	0.81
*ϕ*_LS_	0.19	1.81	0.21	0.21	0.24	0.24
*t*_failing_	*t* = 50 min	Instantaneous	3 h	>48 h	4.5 h	>27 days
Mode of failure	Capillary condensation	Imbibition	Capillary condensation	Did not fail	Capillary condensation	Did not fail
*P* (kPa)	0		103 ± 1		110 ± 1	
*Square (corner radius 3 μm – Sq|3)*						
*θ*_Pr_	90°	90°	142°	140°	139°	137°
*θ*_r_	96° ± 4°	77° ± 2°	137° ± 3°	133° ± 3°	136° ± 1°	129° ± 2°
*θ*_A_	111° ± 2°	83° ± 3°	146° ± 5°	151° ± 1°	153° ± 1°	144° ± 2°
*ϕ*_LV_	0	0	0.89	0.89	0.89	0.89
*ϕ*_LS_	1.88	1.88	0.13	0.13	0.17	0.17
*t*_failing_	Instantaneous	Instantaneous	1.5 h	6 h	3 h	7 h
Mode of failure	Imbibition along corners	Imbibition along corners	Capillary condensation	Failure at corners	Capillary condensation	Failure at corners
*P* (kPa)	–	*–*	96 ± 1		103 ± 1	
*Square (corner radius 50 μm – Sq|50)*						
*θ*_Pr_	90°	90°	135°	132°	133°	130°
*θ*_r_	112° ± 2°	83° ± 5°	134° ± 1°	127° ± 4°	135° ± 2°	120° ± 2°
*θ*_A_	127° ± 3°	85° ± 4°	143° ± 6°	147° ± 1°	151° ± 2°	143° ± 1°
*ϕ*_LV_	0	0	0.84	0.84	0.84	0.84
*ϕ*_LS_	1.79	1.79	0.17	0.17	0.20	0.20
*t*_failing_	Instantaneous	Instantaneous	2 h	27 h	5 h	16 h
Mode of failure	Imbibition along corners	Imbibition along corners	Capillary condensation	Failure at corners	Capillary condensation	Failure at corners
*P* (kPa)	–		100 ± 1		118 ± 1	
*Hexagonal (corner radius 3 μm - Hex|3)*						
*θ*_Pr_	90°	90°	142°	140°	139°	137°
*θ*_r_	98° ± 7°	23° ± 3°	132° ± 2°	125° ± 3°	134° ± 3°	124° ± 3°
*θ*_A_	126° ± 1°	33° ± 2°	149° ± 1°	141° ± 2°	146° ± 1°	139° ± 1°
*ϕ*_LV_	0	0	0.89	0.89	0.89	0.89
*ϕ*_LS_	1.86	1.86	0.13	0.13	0.17	0.17
*t*_failing_	Instantaneous	Instantaneous	3 h	8 h	4 h	9 h
Mode of failure	Imbibition along corners	Imbibition along corners	Capillary condensation	Failure at corners	Capillary condensation	Failure at corners
*P* (kPa)	–	–	106		106	
*Hexagonal (corner radius 50 μm –Hex|50)*						
*θ*_Pr_	90°	90°	139°	136°	136°	133°
*θ*_r_	106° ± 2°	54° ± 1°	132° ± 2°	122° ± 3°	128° ± 2°	124° ± 3°
*θ*_A_	124° ± 2°	61° ± 2°	148° ± 2°	143° ± 2°	141° ± 4°	137° ± 2°
*ϕ*_LV_	0	0	0.87	0.87	0.87	0.87
*ϕ*_LS_	1.44	1.44	0.15	0.15	0.19	0.19
*t*_failing_	Instantaneous	Instantaneous	1.5 h	>48 h	4 h	30 h
*P* (kPa)	–		103 ± 1		120 ± 1	
Mode of failure	Imbibition along corners	Imbibition along corners	Capillary condensation	Did not fail	Capillary condensation	Failure at corners

**Fig. 2:**
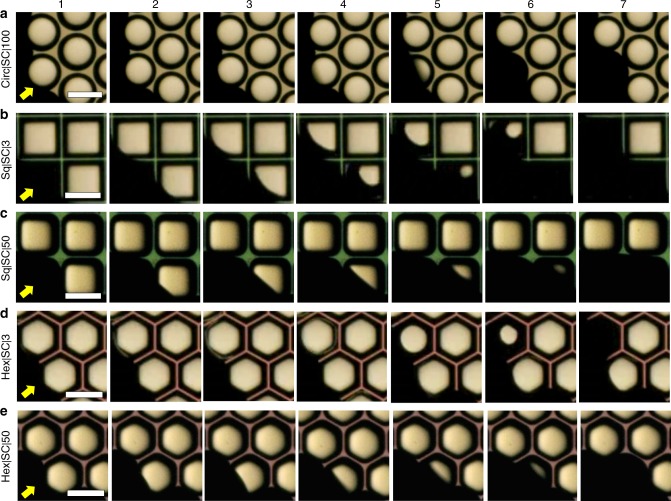
Silica surfaces with simple cavities (SCs) immersed in hexadecane. Series of frames obtained by a high-speed camera at 1500 fps during imbibition of hexadecane in SCs of **a** circular (*D* *=* 200 µm), **b**, **c** square, and **d**, **e** hexagonal geometries of **b**, **d** sharp (*r* = 3 µm) and **c**, **e** rounded (*r* = 50 µm) corners (see Supplementary Movies 1–5). Yellow arrows indicate the direction of the advancing liquid at the onset of immersion. Imbibition was faster in cavities with sharper corners as Sq|3 > Hex|3 > Sq| 50 > Hex|50. (Scale bars: 200 µm)

**Fig. 3:**
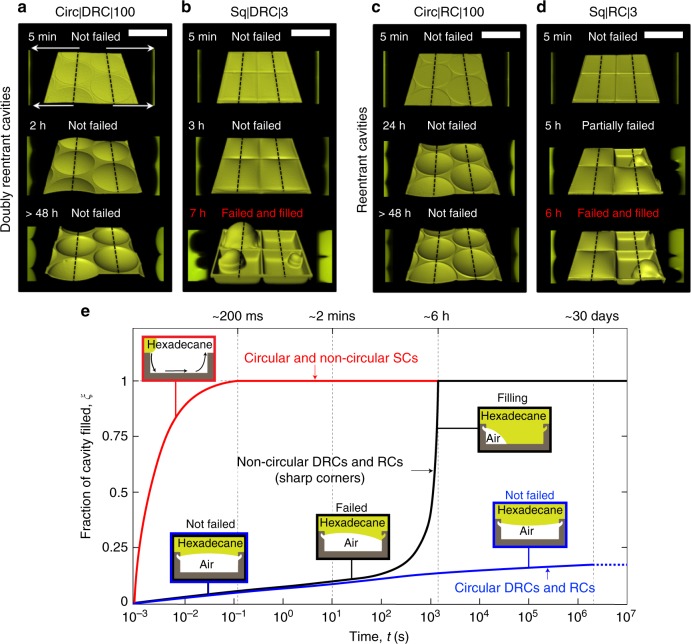
Silica surfaces with reentrant and doubly reentrant cavities immersed in hexadecane. **a**–**d** Computer-enhanced isometric reconstructions of the hexadecane-air interface (center) along with the cross-sectional views along the black dotted lines (on left and right sides of the central image) as a function of time. **a**, **c** Intruding hexadecane menisci were robustly stabilized at the edges of circular DRCs and RCs (Supplementary Figure 8). **b**, **d** In contrast, DRCs and RCs with square shape failed at the corners much faster leading to the loss of trapped air. The stability of the liquid meniscus resting on the edges of the DRCs and RCs was lower if the corners were sharp (Supplementary Figure 7). **e** A semi-quantitative plot demonstrates the efficacy of circular DRCs and RCs in sustaining trapped air for > 3 orders of magnitude longer than square DRCs and RCs, and >7 orders of magnitude longer than commensurate SCs. (Scale bars: 200 µm)

**Fig. 4:**
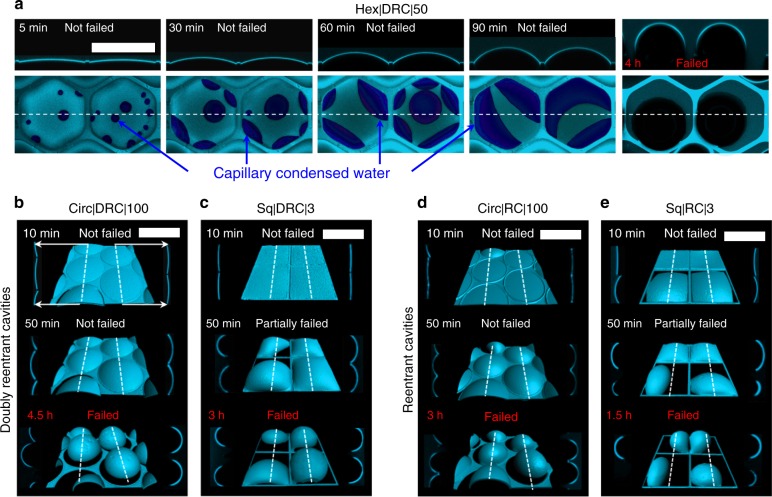
Confocal microscopy images of silica surfaces with reentrant and doubly reentrant cavities immersed in water. **a** Mechanisms underlying the loss of air trapped in a hexagonal DRC on immersion in water and observed via confocal microscopy. The snapshots on top are the cross-sectional views of the top-views below at different times along the white dotted line. The light blue color in the images corresponds to the air-water interface on top that has been rendered partially transparent, while the dark blue color corresponds to the capillary condensed water at the bottom of the cavity. On immersion, the intruding water meniscus was stabilized at the doubly reentrant and reentrant edges. Over next 5–90 min, the capillary condensation of water inside the cavities led to the formation of small droplets that merged over time to form thick films. This volume of water displaced the trapped air causing upward bulging, which initiated the diffusion of trapped air in the water even before *t*_failing_. Eventually the condensed water touched the primary meniscus on top and the cavity was invaded by water. **b**–**e** Computer-enhanced isometric reconstructions (center) along with cross-sectional views along the dotted lines (on either sides of the central image) of representative confocal images of water menisci after immersing silica surfaces with DRCs and RCs of circular and square shapes under a 5 mm high column of water. We observed that circular DRCs and RCs exhibited higher *t*_failing_ than square ones and DRCs outperformed RCs. (Scale bars: 200 µm)

Apparent contact angles (*θ*_r_), advancing contact angles (*θ*_A_), predicted contact angles (*θ*_Pr_), cavity-failing times (*t*_failing_) on immersion, and breakthrough pressures (*P*). Except for cavity-failing times labeled as ‘Instantaneous’ (*t* < 200 ms), which were observed at 1500 fps by high-speed imaging, all others cavity-failing times were recorded via confocal microscopy (Methods). Note: The data in this table are presented in Figs. 2–4, 6, and Supplementary Figures 4, 6, 7, 9, 10

### Investigation with wetting liquids in air

We measured advancing and receding contact angles of drops of hexadecane and water of volume, *V* ≈ 2 μL dispensed at 0.2 μL s^−1^, and found that: (i) silica surfaces with arrays of DRCs and RCs exhibited omniphobicity in air - apparent contact angles for both the liquids, *θ*_r_ > 120°, and (ii) for the surfaces with SCs, the apparent contact angles, *θ*_r_ < 90° for hexadecane and *θ*_r_ ≈ 110° ± 10° for water (Table 1, Supplementary Figure 4). Since the characteristic sizes of the sessile drops were smaller than their capillary lengths, and that the volumes of the air-filled cavities underneath the drops were much lower than the volumes of the drops, we could apply the Cassie-Baxter model to predict apparent contact angles (Supplementary Note 1, 2). We found a reasonable agreement between model predictions, *θ*_Pr_, and the experimental observations for silica surfaces with DRCs and RCs (Table 1 and Supplementary Figure 4). For silica surfaces with SCs, the model predictions, *θ*_Pr_, were corrected for the edge effect^39,40^ (Supplementary Note 3). All cavity microtextures exhibited ultralow receding contact angles, *θ*_R_ ≈ 0°, in stark contrast with doubly reentrant pillars that exhibited superomniphobicity in air^15^. This was due to the presence of crisscrossing connected wetting paths available to receding liquids in the case of cavities, such that liquid meniscus did not undergo periodic detachments from the surfaces, as it does in the case of pillars^41–43^.

### Simple cavities immersed in wetting liquids

Next, we investigated the stability of air trapped in the cavities as the microfabricated silica surfaces were immersed in hexadecane and water. We introduced the liquids at the rate of 2 ml min^−1^ leading to *z* ≈ 5 mm thick columns of liquids above the samples. For the samples with SCs, we imaged wetting transitions at 1500 fps (Supplementary Movies 1–5, Fig. 2). We observed that for both hexadecane and water, SCs of square and hexagonal shapes: (i) got filled within *t* < 200 ms, which we refer to as ‘Instantaneous’ failing in Table 1; (ii) no bubbles were released during these wetting transitions; (iii) the wetting liquids imbibed along the corners, pooled at the bottom, and filled upward pushing the air out; and (iv) cavities with sharper corners started imbibing liquids sooner than those with rounded corners as Sq|3 > Hex|3 > Sq|50 > Hex|50 (Fig. 2 and Supplementary Movies 1–5). Indeed, those results are reminiscent of the rise of wetting liquids in simple capillaries with sharp corners, where liquid columns imbibing along the corners lead the main terminal meniscus in the center (Supplementary Figure 5)^44^. The distance traveled by wetting liquids imbibing along the corners in (semi-infinite) non-circular capillaries, *L*_Imb_, varies as $L_{{\mathrm{Imb}}} \propto k\sqrt {\gamma _{{\mathrm{LV}}}/\mu }$, where *k* empirically correlates with the sharpness of the corners, *γ*_LV_ is the surface tension of the liquid, and *μ* is the dynamic viscosity of the liquid^44^. Thus, imbibition of wetting liquids along the corners determined the fate of the air trapped in SCs.

Contrastingly, in the absence of corners, water could not imbibe into circular SCs. When silica surfaces with circular SCs were immersed in water, air was trapped in the cavities (Supplementary Movie 6). Subsequently, the trapped air was lost within 2 days, observed under cold LED light, via a host of mechanisms, including capillary condensation and diffusion of air in water. When silica surfaces with circular SCs were immersed in hexadecane, they got filled instantaneously due to its lower surface tension and intrinsic contact angle than water (Supplementary Note 3). We expect that smaller SCs might trap air under hexadecane, but this aspect was not explored further.

### DRCs and RCs immersed in a liquid of low vapor pressure

In a dramatic contrast to the SCs, when silica surfaces with arrays of DRCs and RCs, both circular and non-circular, were immersed under *z* ≈ 5 mm thick columns of hexadecane, they trapped air and sustained them for periods ranging from hours to weeks (Table 1). To gain insights into the factors and mechanisms governing the entrapment of air into those cavities at high spatial resolution, we used a laser scanning confocal microscope (Methods, Supplementary Table 1, Supplementary Note 4 and Supplementary Figures 6A, B). Fig. 3 and Supplementary Figure 7 present isometric reconstructions (center) and cross-sectional views (on either sides of the isometric reconstructions) of hexadecane-air interfaces penetrating into DRCs and RCs of different shapes, representative of ~30 cavities of each type. The intruding menisci of hexadecane were rendered flat at the edges of DRCs and RCs, and any further inward push resulted in concave curvatures that prevented the penetration of liquids^15,32^. For the circular cavities, specifically, the hexadecane menisci sagged inside the cavities after *t* = 2 days, though still stabilized at the DRC and RC edges. As a result of sagging, there was an excess capillary pressure on the liquid described by the Young-Laplace relation, $\Delta p \propto \gamma _{{\mathrm{LV}}}/R$, which precluded the diffusion of the trapped air in it (assuming the liquid to be saturated with air), where *γ*_LV_ was the surface tension of the liquid and *R* was the radius of the axis-symmetric curvature of the liquid-vapor interface^45^. Thus, against common expectation, the air trapped inside the circular DRCs and RCs was completely intact even after 27 days (Fig. 3a, c, e and Supplementary Figure 8), a duration that was over seven orders of magnitude longer than for the SCs (Fig. 3e and Table 1); the experiment was discontinued afterwards. These observations underscore the utility of both DRC and RC microtextures in trapping air under wetting liquids of low vapor pressures.

Next, we define *t*_failing_ as the average time taken by a wetting liquid to penetrate beyond the doubly reentrant or reentrant edges and land on the walls of >50% of the cavities (Table 1). We consider *t*_failing_ to be a crucial metric for characterizing the metastability of the trapped air in microtextured surfaces, because for times *t* > *t*_failing_ the specific benefits of the doubly reentrant or reentrant profiles are lost. Subsequently, both doubly reentrant and reentrant cavities behaved as SCs immersed in liquids, which have been studied recently^26,29,46–48^.

When compared with the remarkable stability of the intruding hexadecane menisci at the edges of circular DRCs and RCs, evidenced by high *t*_failing_, the non-circular cavities with similar profiles did not perform as well (Fig. 3 and Supplementary Figure 7 and Table 1). The overall trend was that DRCs and RCs performed similarly - *t*_failing_ varied as Circ|100 ( > 27 days) » Hex|50 (30 h) > Sq| 50 (16 h) > Hex|3 (9 h) > Sq|3 (7 h) (Supplementary Figures 7, 8 and 9). We consider that the sharpness of the corners was proportional to the concentration of mechanical stress at the solid-liquid-gas triple lines^49^, in a somewhat similar fashion to the stress concentration at the corners in solid plates under tension^50^. This stress concentration at the corners could potentially lead to the liquid menisci falling onto the inner walls due to environmental factors, such as mechanical vibrations^51^, or the formation of a conjoining precursor film over time;^52–54^ however, those effects were not considered in this study. At *t* > *t*_failing_, the curvature of the liquid–vapor interface changed its sign and the diffusion of the air trapped inside the cavity into the liquid started^55^ (Fig. 3).

### DRCs and RCs immersed in a liquid of high vapor pressure

**Fig. 5:**
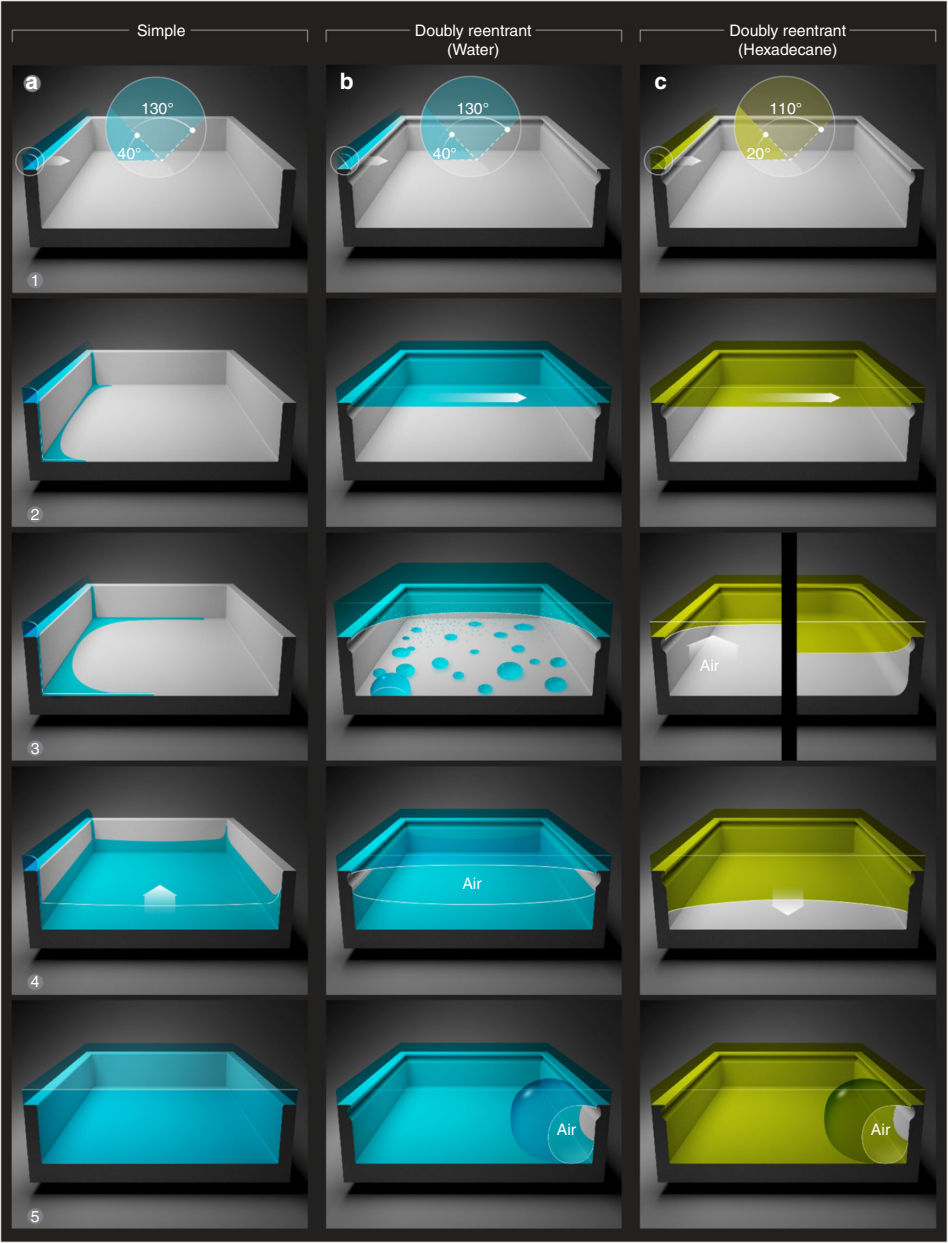
Summary of the filling mechanisms. **a** Wetting liquid spreading over a simple cavity with sharp corners: (1–2) Due to the geometric discontinuity at the edges, the advancing liquid meniscus got pinned (Supplementary Note 3). (3) In parallel, streams of wetting liquid imbibed into the cavities along the corners, which (4) on reaching the base of the cavity formed a film that (5) rose upwards as a column of liquid displacing air. As a result, liquid imbibition along the corners was the primary mechanism of filling in simple non-circular cavities; no bubbles are observed on immersion. **b** DRCs immersed in a wetting liquid of high vapor pressure (water): (1–2) The liquid meniscus was stabilized at the doubly reentrant edge. (3) Water droplets started condensing at the bottom of the cavity, which displaced the trapped air and pushed the primary air-water meniscus upwards; diffusion of air in water began. (4) The condensed droplets agglomerated into a thicker film that grew upwards until (5) it reached the double reentrant edge and merged with the top meniscus leading to partial release of the trapped air as a bubble. **c** DRCs immersed in a wetting liquid of low vapor pressure and low surface tension: (1) and (2) similarly to the case (**b**). (3) Subsequently, if the corners are rounded, the liquid meniscus started sagging (right side); if the corners are sharp, the liquid meniscus ‘failed’ on to the sidewalls before the downward sagging started (left side). Till *t* < *t*_failing_, the trapped air did not diffuse into the liquid (assuming the liquid is saturated with air). (4) At time, *t* > *t*_failing_, the curvature of the liquid meniscus got inverted and molecular diffusion of air into the liquid led to loss of the trapped air

When dealing with wetting liquids of high vapor pressure at temperatures near their dew points, the release of the entrapped air could be driven by capillary condensation (Fig. 4a, Figs. 5B3–5)^56,57^. For instance, when we investigated the stability of air trapped in DRCs and RCs underwater via confocal microscopy, the behavior was dramatically different from that of hexadecane - the rates of wetting transitions depended sensitively on the power of the laser source and the exposure-times (Supplementary Note 4 and Supplementary Figure 6C, D)^51,58–60^. Thus, we chose to use the lowest possible laser intensity, 0.2 mW, and exposure times < 5 min and collected data at intervals of 10 min for the first 2 h and 30 min onwards (Methods). We observed the following qualitative trends (Figs. 4, 5b): (i) the intruding water menisci got stabilized at the edges of DRCs and RCs, (ii) next, the primary water-vapor menisci started bulging upwards due to the capillary condensation of water inside the cavities and the concomitant displacement of the trapped air, (iii) the capillary condensed droplets merged and the water-front grew bottom–upward to eventually touched the primary meniscus stabilized at the doubly reentrant or reentrant edge leading to water caving in, often (iv) accompanied with pinning or the release of air bubbles. We note that the condensation of water inside the cavities displaced the trapped air leading to a build-up of pressure (Figs. 4e, 5b). As a result, the diffusion of gas in water started even at *t* < *t*_failing_. Interestingly, DRCs consistently entrapped air ~2-times longer (3–5 h) than commensurate RCs (1.5–3 h), while the sharpness of the corners did not have much effect (Fig. 4b–e and Supplementary Figure 10). We consider that the extra edge present in the doubly reentrant profiles prevented the merger of the top water meniscus with the capillary condensed waterfront for longer durations in comparison to the commensurate reentrant profiles (Supplementary Figure 11), though it needs experimental verification.

When we investigated the entrapment of air in circular DRCs underwater illuminated by cold white LED light, the entrapment of air lasted for ~9 days (Supplementary Figure 9A). We consider that a coupling between capillary condensation of water and diffusion of trapped air in water drives wetting transitions, but its detailed theoretical analysis in the context of confined cavities^61^ falls beyond the scope of this work.

Next, to assess the significance of intrinsic contact angles and contact angle hysteresis on wetting transitions driven via capillary condensation, we compared our microtextured silica surfaces with two different surface treatments, namely (i) superhydrophilic: *θ*_o_ ≈ 0^°^for water/vapor system obtained via O_2_ plasma treatment (Methods), and (ii) hydrophilic: *θ*_o_ ≈ 40^°^ (Supplementary Note 5 and Supplementary Figure 12). As superhydrophilic silica surfaces (*θ*_o_ = 0°) with arrays of DRCs or RCs were immersed in water, the intruding menisci were stabilized at the edges (explained above), but failed within *t*_failing_ ~1 s, followed by complete filling in *t* ~ 6 s (Supplementary Movie 7). We consider that soon after immersion under water, a continuous film of water formed on the walls of silica cavities driven by capillary condensation due to the near-saturation of the cavities with water vapor within ~1 ms (explained above). The formation of this continuous film of water around the trapped air enabled its release under the effect of buoyancy as more water drained in (Supplementary Figure 13). In contrast, if the same experiment was performed with silica surfaces with intrinsic contact angles for water, *θ*_o_ ≈ 40^°^, the trapped air was released in ~9 days under a *z* ≈ 5 mm column of water (Supplementary Figure 9A). In this scenario - (i) capillary condensation led to the formation of droplets of water instead of films^56,62^, (ii) partial coalescence of droplets led to a patchy water film inside the cavity and due to the high contact angle hysteresis, *θ*_A_ − *θ*_R_ ≈ 40^°^, the pinning forces dramatically exceeded buoyancy (Supplementary Note 6). We also note that due to our strict protocols for sample storage (Supplementary Note 5), the changes in the intrinsic contact angle, *θ*_o_ = 0^°^→40^°^, were due to the partial dehydration of freshly hydroxylated silica surfaces, and not a result of the airborne contamination (Supplementary Figure 12). To summarize, for liquids that cast ultralow intrinsic contact angles, *θ*_o_ ≈ 0^°^ and are prone to capillary condensation, the proposed biomimetic strategy for long-term entrapment of air under wetting liquids would not be feasible.

We summarize the mechanisms underlying the loss of the air entrapped in DRCs and RCs and the displacement of air in SCs by wetting liquids of low and high vapor pressures in Fig. 5.

### Breakthrough pressures of DRCs, RCs, and SCs

**Fig. 6:**
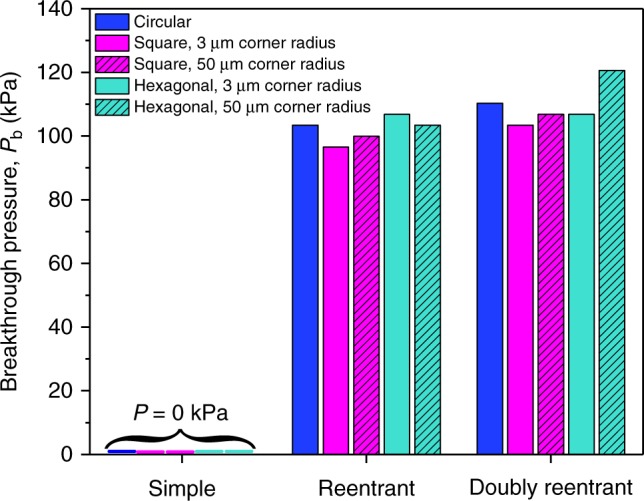
Breakthrough pressures of different microtextures. Breakthrough pressures for SCs, RCs, and DRCs of different shapes and primary dimension, *D* = 200 µm measured with water as the probe-liquid at NTP within seconds after immersion (to minimize the effects of condensation). Experimental error in those measurements: ±1 kPa)

In order to cater to the real-world applications in drag reduction and membrane-based separation processes, the entrapment of air in the microtexture must be as robust as possible. To quantify this aspect, we compared the breakthrough pressures of the different microtextures, defined as the pressure at which the intruding liquid, stabilized at doubly reentrant edges, penetrated >50% of the cavities under observation using a home-built pressure cell (Supplementary Note 7 and Supplementary Figure 14) with water as the probe-liquid. Since the depth of all the cavities considered in this work was 1/4th of the diameter, breakthrough occurred when the primary meniscus touched the cavity floor (Supplementary Figure 15). Based on the statistics from approximately nine cavities of each kind, we found that (i) DRCs exhibited 6–18% higher breakthrough pressures than commensurate RCs, and (ii) within the subgroups of doubly reentrant or reentrant profiles, corners had a minor destabilizing effect as Hex|50 ≈ Circ|100 > Sq|50 ≈ Hex|3 > Sq|3 (Fig. 6, Table 1).

We modeled the breakthrough pressures, *P*_b_, by considering the trapped air to be an ideal gas and its compression as liquid water was pushed inward. For circular DRCs with radius (*R* = 100 μm) and depth *h* ≈ 50 μm, the volumes of the trapped air right after immersion could be approximated as, *V*_1_ = *πR*^2^*h* = 1.6 × 10^6^(μm)^3^ (Supplementary Note 7 and Supplementary Figure 16A). As the liquid is pushed inward, the pressure inside the cavity rises as *P*_2_ = *P*_1_*V*_1_/*V*_2_, where *P*_1_, *P*_2_ and *V*_1_, *V*_2_ are the pressures and volumes of the trapped air right after the immersion (*P*_1_ = 101 kPa) and just before the breakthrough (Supplementary Figure 16 B). We estimated that just before the water meniscus touched the bottom of the cavity (depth, *h*≈50 μm), the entrapped air was compressed to nearly 46% of the original volume, which raised the pressure inside the cavity to *P*_2_ ≈ *P*_1_*V*_1_/*V*_2_ = 2.2*P*_1_, leading to the predicted breakthrough pressure, *P*_b_ = *P*_2_−*P*_1_ = 116 kPa, which is in reasonable agreement with our experiments (See Fig. 6, Supplementary Note 7, and Supplementary Table 2). We also note that Laplace pressure, *P*_L_, due to the curvature of the liquid at doubly reentrant and reentrant edges, also prevents the imbibition as, *P*_L_ = 2*γ*_LV_*C*_m_, where *γ*_LV_ = 72 mN m^−1^ is the surface tension of water, *C*_m_ = 0.5 × (1/*R*_1_ + 1/*R*_2_) is the mean curvature of water-vapor interface and *R*_1_ and *R*_2_ are the two mutually orthogonal radii of curvatures of the water-vapor interface. For circular DRCs under our experimental conditions, we estimate *R*_1_ = *R*_2_ ≈ 125 μm at the time of breakthrough that yields the maximum *P*_L_ ≈ 2 × 72 × 10^−3^ × 1/(125 × 10^−6^) ≈ 1.2 kPa (Supplementary Note 7). Whereas *P*_L_≪*P*_b_ for circular DRCs of diameter *D* = 200 μm, the contribution of Laplace pressure would increase as the diameter of the cavities decreases, for instance for *D* = 100 nm, we predict the breakthrough pressure to be *P*_b_≈1700 kPa with nearly 80% contribution from the *P*_L_. Those considerations might enable rational design of DRCs for specific applications.

## Discussion

In order to achieve long-term entrapment of air at solid–liquid interfaces for drag reduction, current approaches rely heavily on perfluorinated coatings that are vulnerable to harsh physical, chemical, and mechanical conditions. In response, we investigated the efficacy of biomimetic microtextures composed of doubly reentrant and reentrant cavities that trap air on immersion in wetting liquids, without solely relying on chemical modifications. Remarkably, when silica surfaces with arrays of circular DRCs and RCs were immersed in hexadecane (intrinsic contact angle, *θ*_o_ ≈ 20^°^), the cavities stabilized intruding menisci such that the trapped air remained completely intact even after 27 days, after which the experiment was discontinued. In stark contrast, silica surfaces with arrays of circular or non-circular SCs got filled under hexadecane within *t* ≈ 0.2 s, which was a factor of ~10^7^ shorter. Since the diffusion of air trapped in DRCs and RCs immersed in low vapor pressure liquids starts at *t* > *t*_failing_, it would be prudent to control *t*_failing_ as much as possible by careful design. While we found that DRCs exhibit higher breakthrough pressures than RCs, the complexity of the procedures to achieve DRCs in comparison to RCs indicates that the latter might be the pragmatic choice for scale-up. As explained above, higher breakthrough pressures can be achieved by reducing the diameter, *D*, of cavities (Fig. 1). The quantification of drag reduction exploiting the proposed approach warrants an in depth experimental and theoretical investigation that is beyond the scope of this report.

We also note that our use of microfabrication techniques in this work was only to demonstrate the proof of concept. To create similar biomimetic microtextures on common materials, such as plastics and metals, for the real-world applications, innovative and scalable approaches will be needed; some recent examples include, injection molding^63^, photofluidization^64^, electric discharge machining^31^, femtosecond lasers^65^, and microfluidic emulsion templating^66^. Additional challenges, such as biofouling and scaling, might also affect the topography-based wetting characteristics we observed here. However, to address those challenges, preventive/preemptive chemical treatments could be applied more frequently due to the absence of vulnerable coatings in our approach. For liquids with high vapor pressure, capillary condensation inside the cavities could compromise the long-term entrapment of air. Thus, strategies for removing condensed liquids should be explored. In fact, some of the approaches for replenishing the air trapped in submerged surfaces exploiting phobic coatings could be applicable to this coating-free approach^3,4^, including electrolysis^67^, thermal^68^, pneumatic^69^, and geometry^70^. To conclude, we hope that the insights offered here might unlock the potential of common materials for the development of coating-free surfaces for applications that require immersion of solid surfaces in wetting liquids, such as frictional drag reduction and desalination.

## Methods

### Microfabrication

To fabricate our samples with different profiles, we used silicon wafers (4-inch diameter, <100> orientation and with 2.4 µm thick thermal oxide layer from Silicon Valley Microelectronics). For the microfabrication process, we applied a 1.6 µm layer of AZ-5214 photoresist on the wafers by spin-coating. The required micro-patterns were designed using Tanner EDA L-Edit software and transferred onto the wafers in a Heidelberg Instruments µPG501 direct-writing system. The UV-exposed photoresist was removed in a bath of AZ-726 developer. The exposed SiO_2_ top layer was etched in an inductively-coupled plasma reactive ion etching (ICP-RIE) equipment by Oxford Instruments (pressure of 10 mT, RF power at 100 W, ICP power at 1500 W, C_4_F_8_ at 40 sccm and O_2_ at 5 sccm, at *T* = 10 °C, for 13 min). The wafers were then transferred to a Deep ICP-RIE (Oxford Instruments) to etch the Si under the SiO_2_ layer. Below, we explain the specific details of the processes required for microfabricating arrays of simple, reentrant and doubly reentrant cavities.

### Simple cavities

To etch Si we used an anisotropic etching method, or Bosch process, characterized by a sidewall profile control using alternating deposition of a C_4_F_8_ passivation layer (pressure of 30 mT, RF at 5 W, ICP at 1300 W, C_4_F_8_ at 100 sccm and SF_6_ at 5 sccm, at *T* = 15 °C for 5 s) and etching with SF_6_ (pressure of 30 mT, RF at 30 W, ICP at 1300 W, C_4_F_8_ at 5 sccm and SF6 at 100 sccm, at *T* = 15 °C for 7 s). In order to achieve depths of *h* ≈ 50 µm, we cycled this process 184 times.

### Reentrant cavities

For reentrant profiles we used the anisotropic etching recipe for 5 cycles to create a shallow indentation in the Si underneath the SiO_2_ layer. After a piranha-cleansing step, to remove the remnants of the passivation layer in the anisotropic process, we created the void space under the SiO_2_ layer by etching in all directions using an isotropic etching step (pressure of 35 mT, RF at 20 W, ICP at 1800 W, SF_6_ at 110 sccm, at *T* = 15 °C for 125 s). The cavities where then deepened to *h* ≈ 50 µm, by cycled 160 times the already mentioned anisotropic etching process.

### Doubly reentrant cavities

For doubly reentrant profile we used the same anisotropic etching method, cycled 5 times to create a small indentation in the Si. After a piranha-cleansing step to remove the remnants of the passivation layer, an isotropic etching step was performed (pressure of 35 mT, RF at 20 W, ICP at 1800 W, SF_6_ at 110 sccm, at *T* = 15 °C for 25 s). Next, a 500 nm layer of thermal oxide was grown using a Tystar furnace system. The top and bottom layers of thermal oxide were subsequently etched using the same recipe as in the first SiO_2_ etching step. The next steps included a repetition of the 5 cycles of the anisotropic process used before, a piranha cleanse, followed by the isotropic step described earlier (125 s), to create the void behind the added sidewall of thermal oxide, which then formed the doubly reentrant rim at the edge of the cavity. The final step deepened the cavities up to *h* ≈ 50 µm, using the same anisotropic recipe for 160 cycles.

### Protocols for cleaning and storing the samples

After microfabrication we cleaned the SiO_2_/Si (silica) surfaces with fresh piranha solutions (H_2_SO_4_: H_2_O_2_ = 4:1 at *T* = 388 K) for 10 min, blow-dried with a 99% pure N_2_ pressure gun and stored in glass petri dishes in a dedicated vacuum oven at *T* = 323 K, until the intrinsic contact angle of smooth SiO_2_/Si stabilized to, *θ*_o_ ≈ 40° (after 48 h). Subsequently, the samples were stored in a N_2_ cabinet until needed for characterization.

### Advancing/receding contact angles in air

The static and advancing/receding contact angle measurement using de-ionized water and hexadecane were performed in a Kruss Drop Shape Analyzer - DSA100 at 0.2 μL s^−1^. All the data were analyzed using the Advance software. The error bars in the reported contact angles data are based on the standard deviation of 5–10 measurements.

### High-speed imaging

For samples with SCs the edge of the advancing meniscus (1 ml min^−1^) was filmed at 1500 fps using Edgetronic high-speed camera attached to Qioptics objective with a focal distance of 9.5 cm.

### Scanning electron microscopy

Samples were cleaved using a diamond-tip scriber and coated with a 4 nm Au/Pd layer to minimize electrical charging during SEM (FEI Quanta 600).

### Confocal microscopy

A Zeiss LSM710 upright confocal microscope was used to visualize cavity-filling employing diluted 0.01 M solutions of Rhodamine B (Acros) as fluorescent dye for water experiments and Nile Red (Aldrich) for hexadecane. After fixing the sample at the bottom of a petri dish, the fluorescent solution was gently poured sideways until the sample was completely covered by a *z* ≈ 5 mm column of solution. A 20X immersion objective was then lowered to the working distance and the experiments where immediately started. Sequential images (1024px × 1024px) were taken in the *Z*-stack mode, in which several confocal images were taken from the bottom of the cavities up to 100 microns above the top surface. The intensity of the laser was kept as low as possible - it was 0.2 mW for Rhodamine/water solution and 2 mW for Nile red/hexadecane solution. Subsequently, using the Imaris v.8.1 software, by Bitplane, we performed 3D rendering to obtain isometric images of the liquid-vapor interfacecs and their cross sections to visualize wetting transitions.

### Plasma ashing

O_2_ plasma ashing was carried out in a Diener Electronics plasma system (Atto model) at 200 W for 10 min using ultrapure (99.999%) O_2_ gas supply with a flow 16.5 sccm in a chamber maintained at 300 mTorr pressure.

## Electronic supplementary material

Supplementary Information

Description of Additional Supplementary Files

Supplementary Movie 1

Supplementary Movie 2

Supplementary Movie 3

Supplementary Movie 4

Supplementary Movie 5

Supplementary Movie 6

Supplementary Movie 7

## Data Availability

The authors declare that the data supporting the findings of this study are provided in the article and its Supplementary Information.
