# Supplementary material for: Biomimetic coating-free surfaces for long-term entrapment of air under wetting liquids
Source: Nat Commun. 2018 Sep 6;9:3606. doi: 10.1038/s41467-018-05895-x (PMC6127334; doi:10.1038/s41467-018-05895-x)
Supplement: Supplementary file 1 — Supplementary Information [file 41467_2018_5895_MOESM1_ESM.pdf]

Supplementary Information

Biomimetic coating-free surfaces for long-term entrapment of air under wetting liquids

Eddy M. Domingues[§], Sankara Arunachalam[§], Jamilya Nauruzbayeva & Himanshu Mishra[*]

King Abdullah University of Science and Technology (KAUST), Water Desalination and Reuse Center (WDRC), Biological and Environmental Sciences and Engineering (BESE) Division, Thuwal 23955-6900, Saudi Arabia

[§]Equal contribution
[*]Corresponding author: himanshu.mishra@kaust.edu.sa

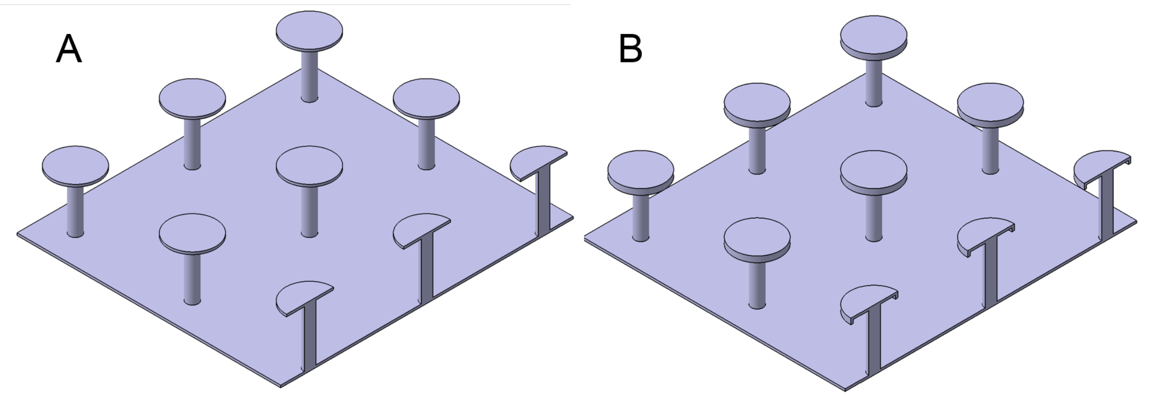

Supplementary Figure 1. A schematic representation of pillars. (A) Reentrant pillars **(B)** Doubly reentrant pillars.

Supplementary Note 1: Calculations of cavity volume and capillary lengths of probe liquids

All the samples investigated in this study had an array of cavities with the primary dimension of D = 200 μm, pitch, L = 212 μm and depth of $h \approx 50$ μm. We estimated the volume of the cavities by estimating its base area and multiplying by its depth, h, as shown in Supplementary Figure 2. For example, in the case of circular cavity, the volume is equal to,

$$V_{\mathrm{Cav}} = \pi\, D^2 h/4 \tag{1}$$

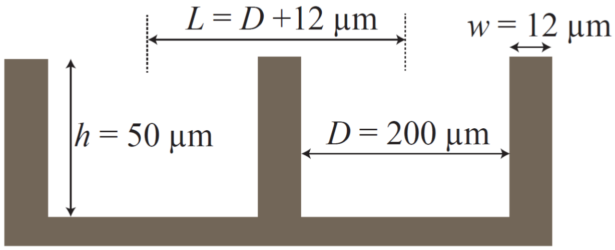

Supplementary Figure 2. A schematic representation of the cross-section of simple circular cavities.

A typical 2 μL water droplet with a 1 mm diameter could cover approximately 18 cavities of primary dimension of D = 200 μm, pitch, L = 212 μm. Since the ratio of the volume of a single cavity to a 2 μL liquid drop was ~1/1270, we could apply the Cassie-Baxter model (Supplementary Note 2).

Next, we calculate the capillary length, λ_{c}, that characterizes the length scale at which interfacial tensions exceed inertial forces [1]:

$$\lambda_{\mathrm{c}} = \sqrt{\gamma_{\mathrm{LV}}/\rho g} \tag{2}$$

where γ_{LV} is the surface tension, ρ is density and g is the acceleration due to gravity. The diameters of the liquid drops employed in our experiments were smaller than the respective capillary lengths (Supplementary Table 1).

Supplementary Table 1: Physical properties of probe liquids at 293 K and 1 atm [2-6].

Liquid	Surface tension, γ_{LV} (mN m^{-1})	Vapor pressure at NTP, p_v (kPa)	Ostwald coefficient for the solubility of N_2 in the liquid, L	Intrinsic contact angle on silica in air, θ_o	Density, ρ (kg m^{-3})	Dynamic viscosity, μ (mPa.s)	Capillary length, λ_c (mm)
Water	72	2.3	0.016	40°	997	0.876	2.7
Hexadecane	28	0.01	0.118	20°	773	3.039	1.9

Supplementary Note 2: The Cassie-Baxter model

Since the volume of the cavities underneath the liquid drops of our microtextured silica surfaces were much smaller than the volumes of the drops themselves, we could employ the Cassie-Baxter model[7] to predict the apparent contact angles,

$$\cos\theta_{\mathrm{Pr}} = \phi_{\mathrm{LS}}\cos\theta_{\mathrm{o}} - \phi_{\mathrm{LV}} \qquad (3)$$

where θ_{Pr} is the apparent contact angle and θ_{o} is the intrinsic contact angle of the liquid on a smooth and flat surface (Young's contact angle)[1]. Here $\phi_{\mathrm{LS}} = A_{\mathrm{LS}}/A_{\mathrm{H}}$ and $\phi_{\mathrm{LV}} = A_{\mathrm{LV}}/A_{\mathrm{H}}$ are the ratios of real liquid-solid (A_{LS}) and liquid-vapor (A_{LV}) areas (Supplementary Figure 3A) compared to the projected horizontal area (A_{H}) (Supplementary Figure 3B).

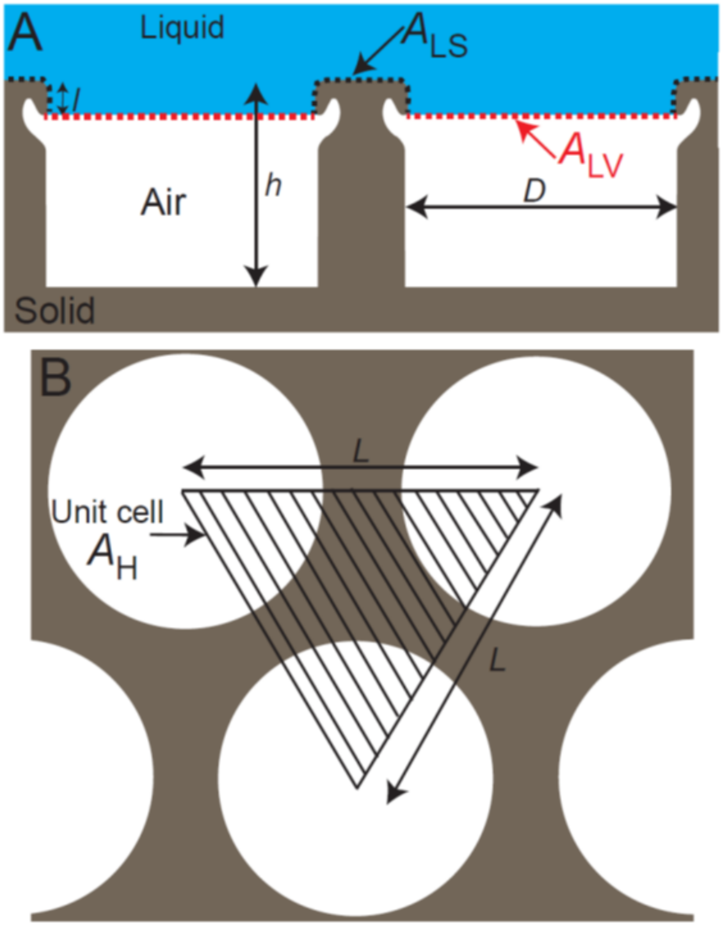

Supplementary Figure 3. A schematic representation of doubly reentrant circular cavities. (A) Cross-section of cavities and a liquid meniscus stabilized at the doubly reentrant edge. **(B)** A unit cell showing projected area for the calculation of ϕ_{LS} and ϕ_{LV}.

Based on the Supplementary Note 1 above and Supplementary Figure 3, ϕ_{LV} and ϕ_{LS} were calculated (in this case for circular cavities) as;

$$\phi_{\mathrm{LV}} = \frac{\pi D^2}{4L^2 \sin 60°} \qquad (4)$$

and

$$\phi_{\mathrm{LS}} = 1 - \frac{\pi D^2}{4L^2 sin60°} + \frac{\pi D l}{L^2 sin60°} \qquad (5)$$

where D is the diameter of the cavity, L is the pitch, l is the height of the reentrant or doubly reentrant rim. We also note that $\phi_{\mathrm{LV}} \geq 0$ and $\Omega \geq \phi_{\mathrm{LS}} \geq 0$, where Ω is the roughness of the microtexture, defined as the total surface area divided by the projected area. For further details on the derivation of Supplementary Equations 4, 5 and definitions of ϕ_{LV} and ϕ_{LS}, please refer the article by Kaufman and co-workers [8].

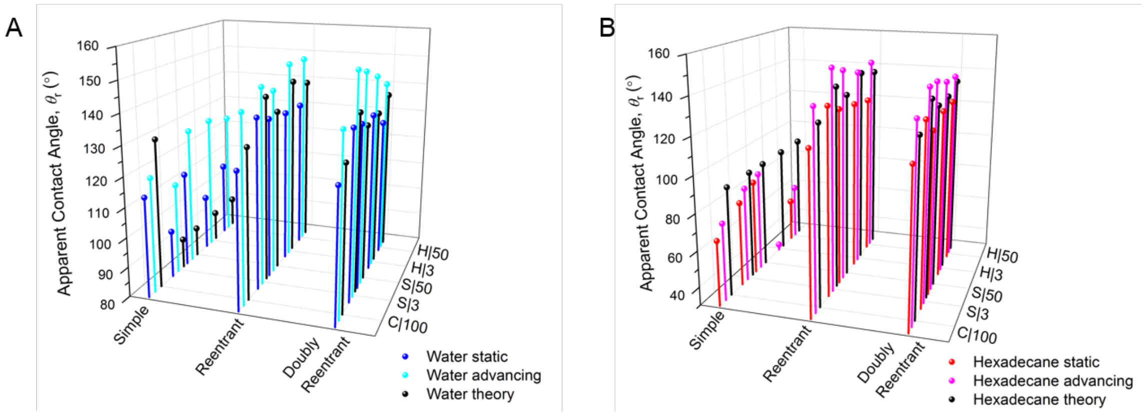

Supplementary Figure 4. Contact angles on silica surfaces with simple, reentrant and doubly reentrant cavities. Static, advancing and theoretically calculated contact angles for **(A)** water and **(B)** hexadecane on. Black dots represent predicted equilibrium apparent contact angles obtained from the Cassie−Baxter model (θ_o = 40° for water and θ_o = 20° for hexadecane). The receding contact angles for both water and hexadecane were less than 5° for all the microtextured surfaces. (Also see Table 1 in the main manuscript)

Supplementary Note 3: The effect of sharp edges on contact angles.

When a liquid is spreading over a flat surface with sharp 90° geometric turns, like our silica surfaces with arrays of simple cavities, there is a ~ 90° increase in the apparent contact angles at the edges of those features, known as the edge effect (Figures 5A1,B1, C1) [9,10]. During immersion in water, as water spread over silica surfaces with arrays of simple circular cavities, the combination of intrinsic contact angle ($\theta_o \approx 40^{\circ}$), primary dimension, D = 200 μm, and the edge effect enabled the entrapment of air in the cavities. In contrast, for hexadecane, lower intrinsic contact angle and surface tension, in comparison to water (Supplementary Table 1), led to the instantaneous filling of even simple circular cavities. Though, we consider that as the primary dimension of the circular cavities is reduced further, even hexadecane meniscus might trap air underneath due to the edge effect; this issue warrants further experimental investigation.

While modeling apparent contact angles on silica surfaces with arrays of simple cavities, that got filled instantaneously with wetting liquids, the predicted contact angles were corrected for the edge effect by adding 90° to θ_{Pr} (Table 1). On the other hand, predictions of the Cassie-Baxter model (Supplementary Note 2) were higher than the observed apparent contact angles for the simple circular cavities with water (Table 1). We consider that it was due to the filling of cavities at the edge of the drop over time [8,11-13].

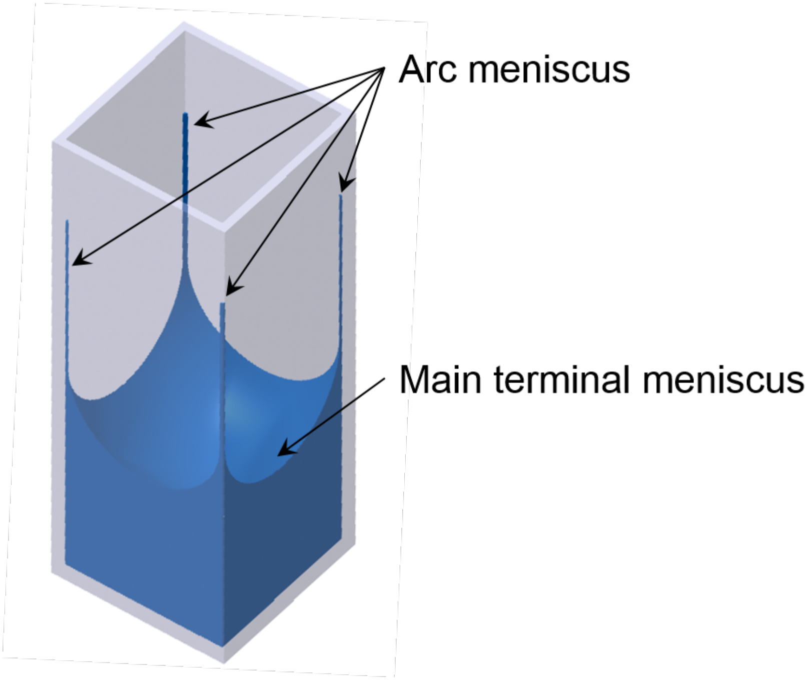

Supplementary Figure 5. Liquid imbibition along corners. A schematic representation of imbibition of a wetting liquid along the corners of an intrinsically wetting capillary of square cross-section. Arc menisci, along the corners, rise faster and higher than the main terminal meniscus.

Supplementary Note 4: Confocal Microscopy

A Zeiss LSM710 upright confocal microscope was used to visualize cavity-filling mechanisms employing diluted 0.01 M solutions of Rhodamine B (Acros) as fluorescent dye for water experiments and Nile Red (Aldrich) for hexadecane. After fixing the sample at the bottom of a petri dish, the fluorescent solution was gently poured sideways until the sample was completely covered by a $z \approx 5$ mm column of solution. A 20X immersion objective was then lowered to the working distance and the experiments where immediately started. Sequential images (1024px ×1024px) were taken in the *Z-stack* mode, in which several confocal images were taken from the bottom of the cavities up to 100 microns above the top surface. The intensity of the laser was 0.2 mW for Rhodamine/water solution and 2 mW for Nile red/hexadecane solution for optimum imaging. Subsequently, using the Imaris v.8.1 software, by Bitplane, we were able to make 3D rendered surfaces and several cross sections to help visualize the wetting transition in our cavities.

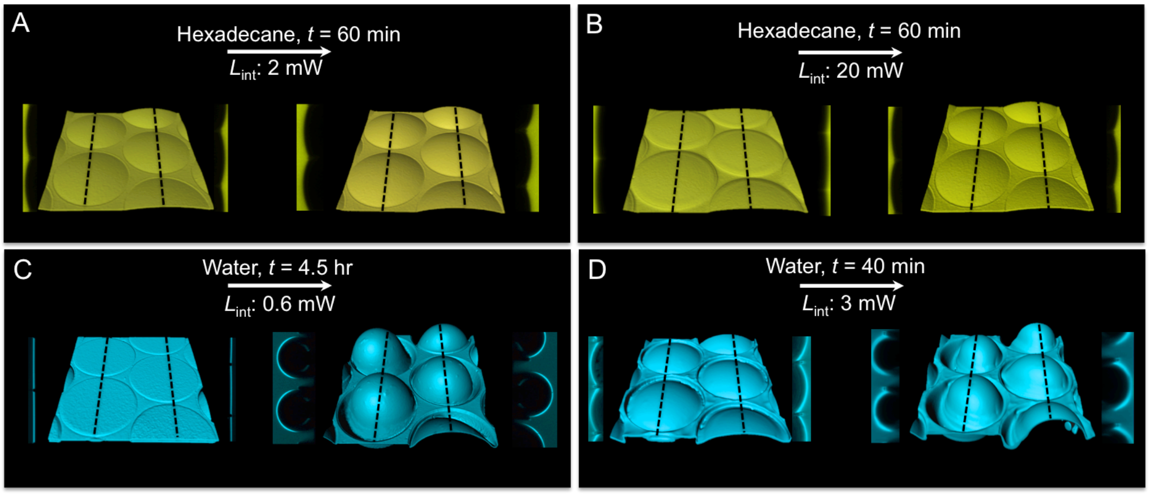

Supplementary Figure 6. **Effects of laser power and exposure intervals on $t_{failing}$ of doubly reentrant cavities observed via confocal microscopy.** Figures **A** and **B** demonstrate that due to the ultralow vapor pressure of hexadecane, increasing laser power from 2 mW to 20 mW did not affect $t_{failing}$. Contrastingly, the wetting transition was faster when the same samples were immersed in water. Figures **C** and **D** demonstrate cavity filling underwater, when we increase the laser power from 0.6 mW to 3 mW (a factor of 5), wetting transitions from partially- to fully-filled states reduced from 4.5 h to 40 minutes. Thus, our simple experiments demonstrate that for liquids with high vapor pressure, capillary condensation drives cavity filling, whereas for liquids with low vapor pressures, it is not a key mechanism.

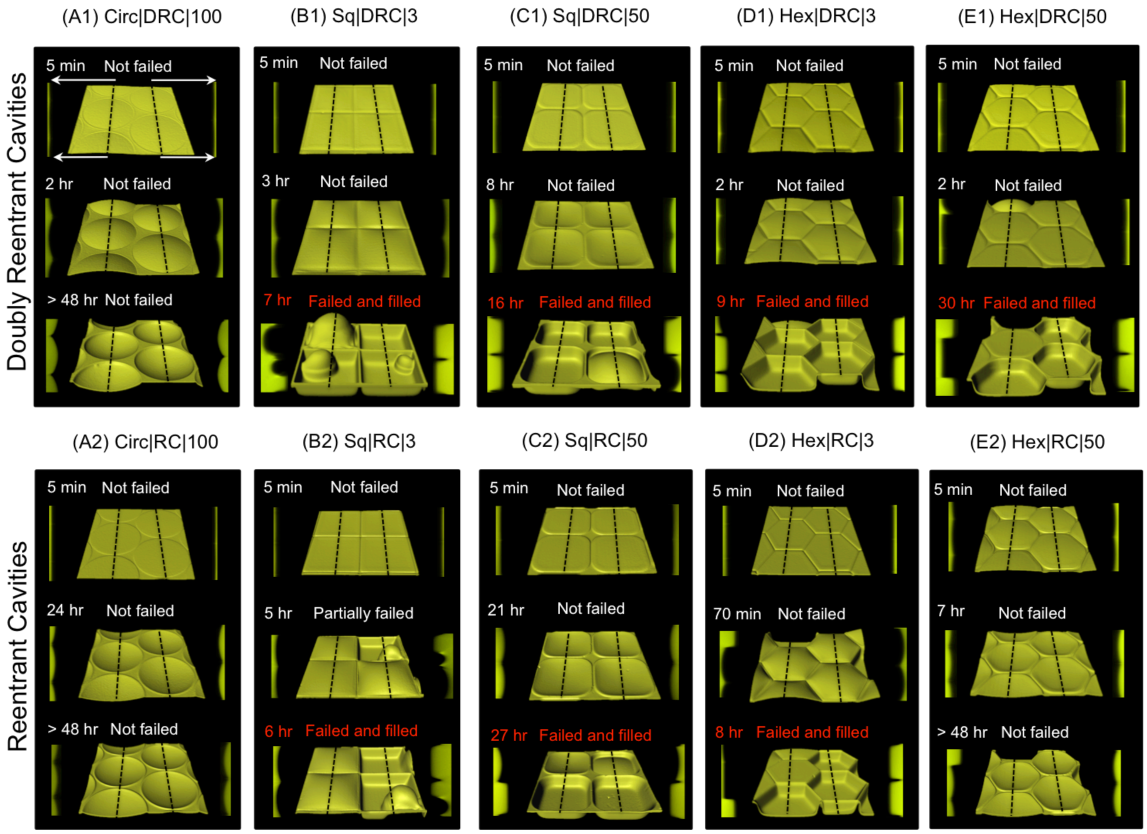

Supplementary Figure 7. Confocal microscopy images of microtextured silica surfaces under hexadecane. Computer-enhanced 3D reconstructions of representative confocal images (top-views and cross-sections along the dotted lines) of wetting transitions in silica surfaces with doubly reentrant and reentrant cavities immersed under a $z \approx 5$ mm column of hexadecane (intrinsic contact angle on silica in air, $\theta_o \approx 20^{\circ}$). The (false) yellow color in the images corresponds to the interfaces of hexadecane with the trapped air and the solid surface underneath. Images on left and right of the central 3D images are the cross sections of the 3D surface taken along the black dotted lines. (Scale bars: edge length for squares is 200 µm, diameter for circles is 200 µm, and distance between opposite sides in the hexagons is also 200 µm)

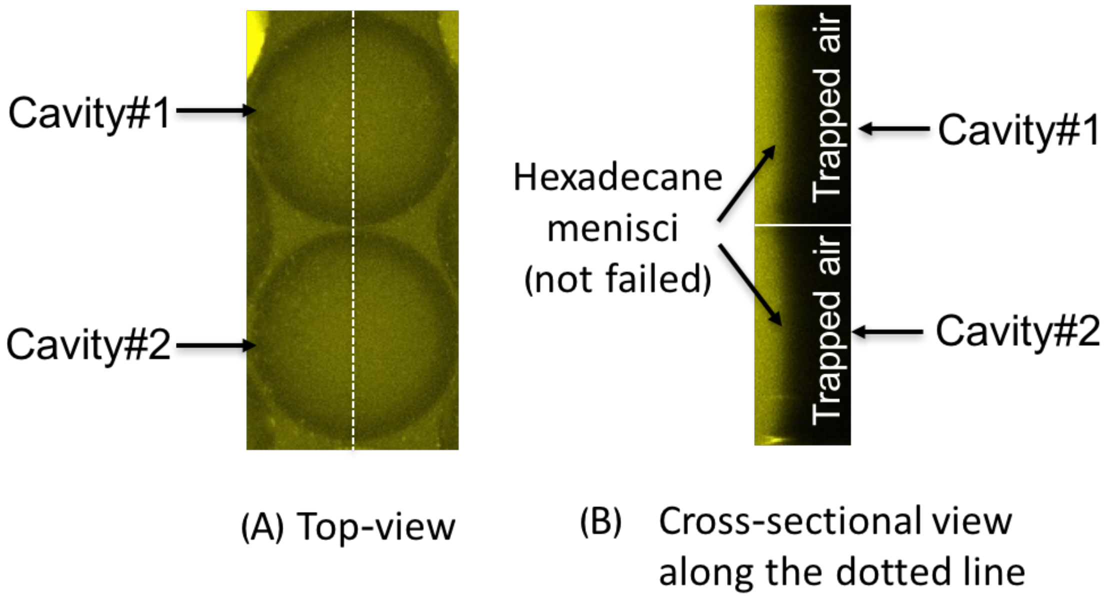

Supplementary Figure 8. Confocal microscopy images of circular doubly reentrant cavities under hexadecane. Computer enhanced confocal microscopy images of silica surfaces with arrays of circular doubly reentrant cavities, Circ|DRC|100, after 27 days of immersion in a $z \approx 5$ mm thick column of hexadecane. **(A)** Top view, **(B)** cross-sectional view of along the white dotted line. The images clearly demonstrate that the liquid meniscus is still stabilized at the DRC edge. (Scale bar: Cavity diameter is 200 μm)

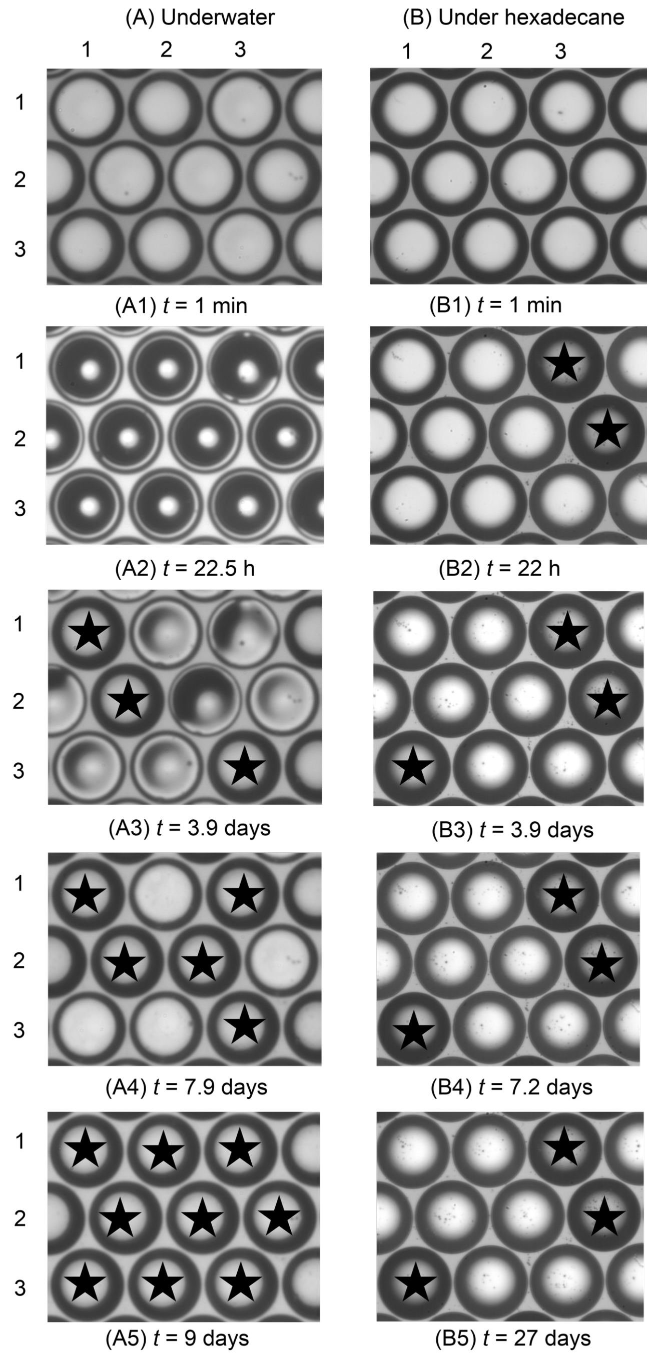

Supplementary Figure 9. **Optical micrographs of silica surfaces with arrays of circular doubly reentrant cavities under wetting liquids.** Silica surfaces with Circ|DRC|100, underwater (intrinsic contact angle on silica in air, $\theta_o \approx 40^{\circ}$) and under hexadecane ($\theta_o \approx 20^{\circ}$). The filled cavities are marked with the star (★) symbol. The cavities underwater got filled after 9 days, mainly due to the condensation of water, diffusion of trapped air and the merger of the condensed water and the primary meniscus on top. In the case of hexadecane, the majority of cavities trapped air for a much longer time (we observed the samples up to ~27 days, but air was still trapped). (Scale: D = 200 μm)

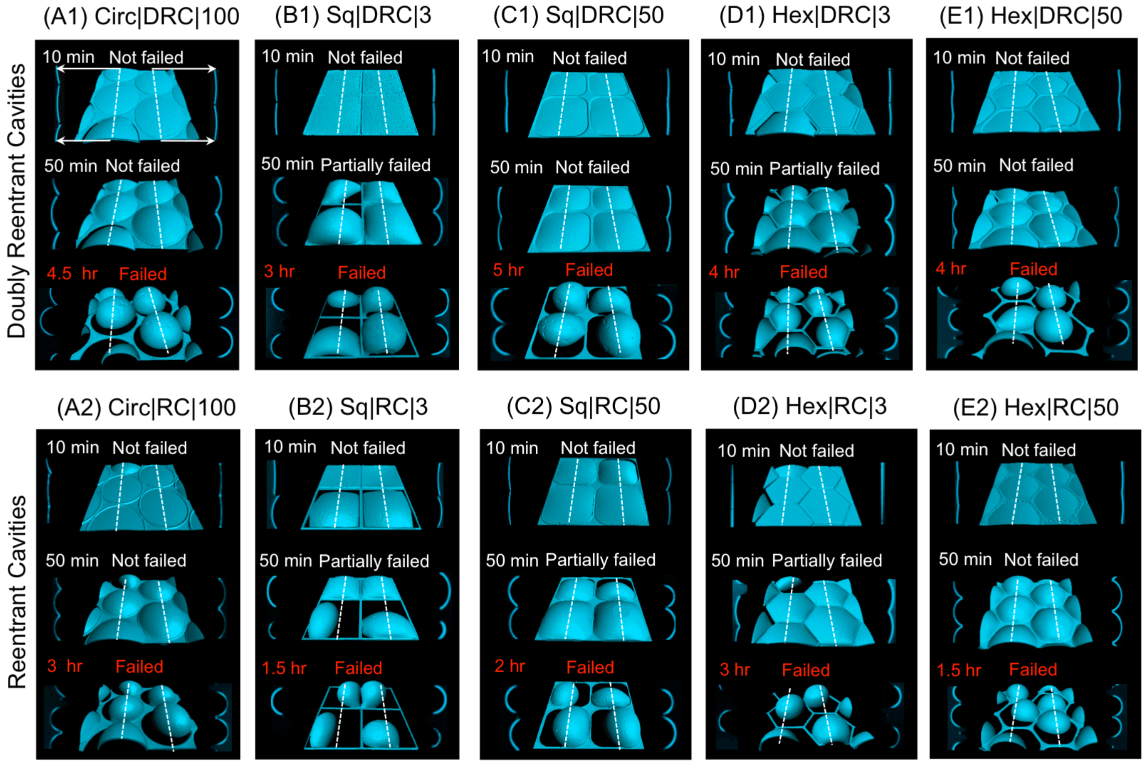

Supplementary Figure 10. Confocal microscopy images of microtextured silica surfaces underwater. Computer-enhanced isometric reconstructions (center) along with cross-sectional views along the dotted lines (on either sides of the central image) of representative confocal images of water menisci after immersing silica surfaces with DRCs and RCs of various shapes under a 5 mm high column of water. The (false) blue color in the images corresponds to the interfaces of water with the trapped air and the solid surface underneath. We observed that water menisci remained flat on top of cavities stabilized at doubly reentrant edges for first few minutes. Subsequently, after ~1 hr of immersion, primary liquid menisci started to bulge up noticeably. In some cavities, liquid penetrated through the corners. (Scale bars: the diameter of circular cavities is 200 μm, edge length for squares is 200 μm and distance between opposite sides in the hexagons is also 200 μm)

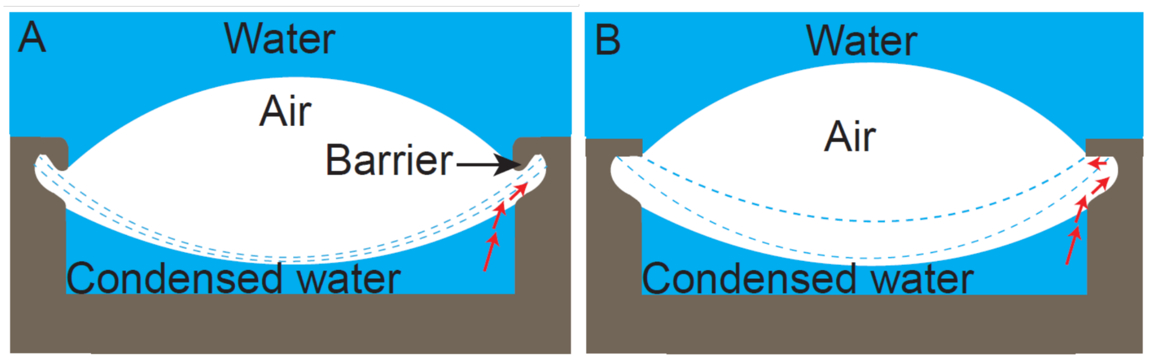

Supplementary Figure 11. **Capillary condensation in doubly reentrant and reentrant cavities.** A schematic representation of the capillary condensed film of water growing bottom-upward in intrinsically wetting **(A)** doubly reentrant cavities, and **(B)** reentrant cavities, immersed in water. The presence of an extra barrier for the doubly reentrant cavities **(A)** prevented the merger of the condensed water and the liquid meniscus on top for longer timescales than reentrant cavities **(B)**.

Supplementary Note 5: Protocols for cleaning and storing the samples

After microfabrication we cleaned the SiO_2/Si (silica) surfaces with fresh piranha solutions (H_2SO_4 : H_2O_2 = 4:1 at T = 388 K) for 10 min, blow-dried with a 99% pure N_2 pressure gun and stored in glass petri dishes in a dedicated vacuum oven at T = 323 K, until the intrinsic contact angle of smooth SiO_2/Si stabilized to, $\theta_o \approx 40°$ (after 48 h). Subsequently, the samples were stored in a N_2 cabinet until needed for characterization.

We performed X-ray photoelectron spectroscopy (XPS) on silica samples (i) freshly cleaned by piranha solution, (ii) after 24 in vacuum, (iii) 48 h in vacuum, and (iv) 48 h in vacuum followed by one week of storage in a N_2 cabinet (all stored in glass petri dishes), as shown in Supplementary Figure 12. We found no significant evidence of airborne hydrocarbon impurities or perfluorocarbon residues from the etching process. We concluded that the increase in the intrinsic contact angle of silica surfaces to the water/vapor system, $\theta_o \approx 0^\circ \rightarrow 40^\circ$, was due to the partial dehydroxylation of such surfaces (Methods).

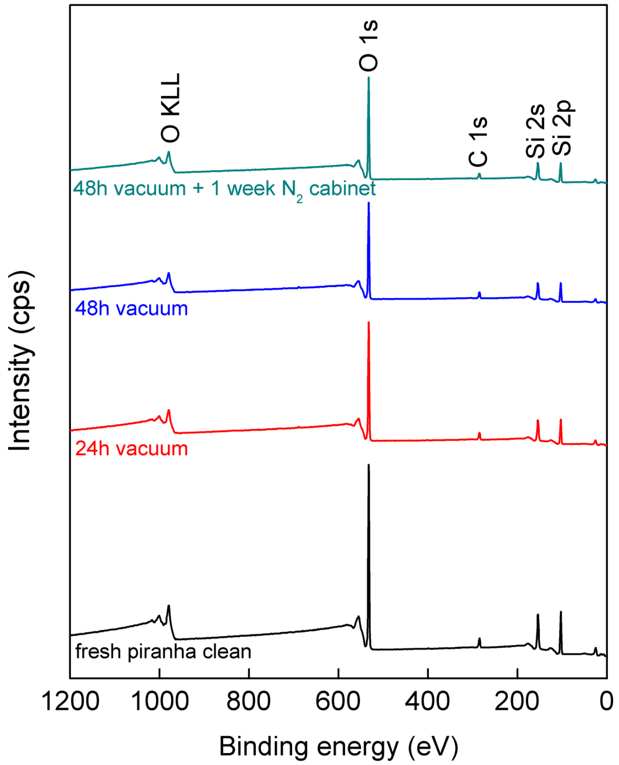

Supplementary Figure 12. **X-ray photoelectron spectroscopy (XPS) characterization**. XPS spectra on silica samples freshly cleaned by piranha solution, after 24 in vacuum, 48 h in vacuum and 48 h in vacuum followed by one week of storage in N_2 cabinet in glass petri dish.

Supplementary Note 6: Pinning and depinning of bubbles

A simple comparison of the pinning forces at the mouths of doubly reentrant circular cavities,

$$F_{\mathrm{Pin}} \sim \gamma_{\mathrm{LV}} \times \pi D \times (cos\theta_{\mathrm{R}} - cos\theta_{\mathrm{A}}) \qquad (11)$$

against the buoyancy of the bubble,

$$F_{\mathrm{Buoy}} \sim V_{\mathrm{Cav}} \times (\rho_{\mathrm{L}} - \rho_{\mathrm{G}}) \times g \qquad (12)$$

shows that buoyancy is insignificant at this length scale ($F_{\mathrm{Pin}}/F_{\mathrm{Buoy}} \approx 10^2$), where $\theta_{\mathrm{A}} \approx 40^{\circ}$ and $\theta_{\mathrm{R}} \approx 0^{\circ}$ were the advancing and receding contact angles of water on silica [14]. The estimated pinning force experienced by the trapped bubble at the mouth of the cavity was significantly higher than buoyancy.

In the case of O_2 plasma treated silica surfaces, the intrinsic contact angles of water in air were, $\theta_{\mathrm{o}} \approx 0^{\circ}$. This was due to the highly hydroxylated surface of the silica after plasma activation (Methods). As a result, condensation of water led to formation of continuous films instead of discrete drops [15]. After the film spread across the cavity, the main meniscus collapsed inward asymmetrically, and the trapped bubble rose out of the cavity due to buoyancy and the absence of pining force (Supplementary Figure 13A-D).

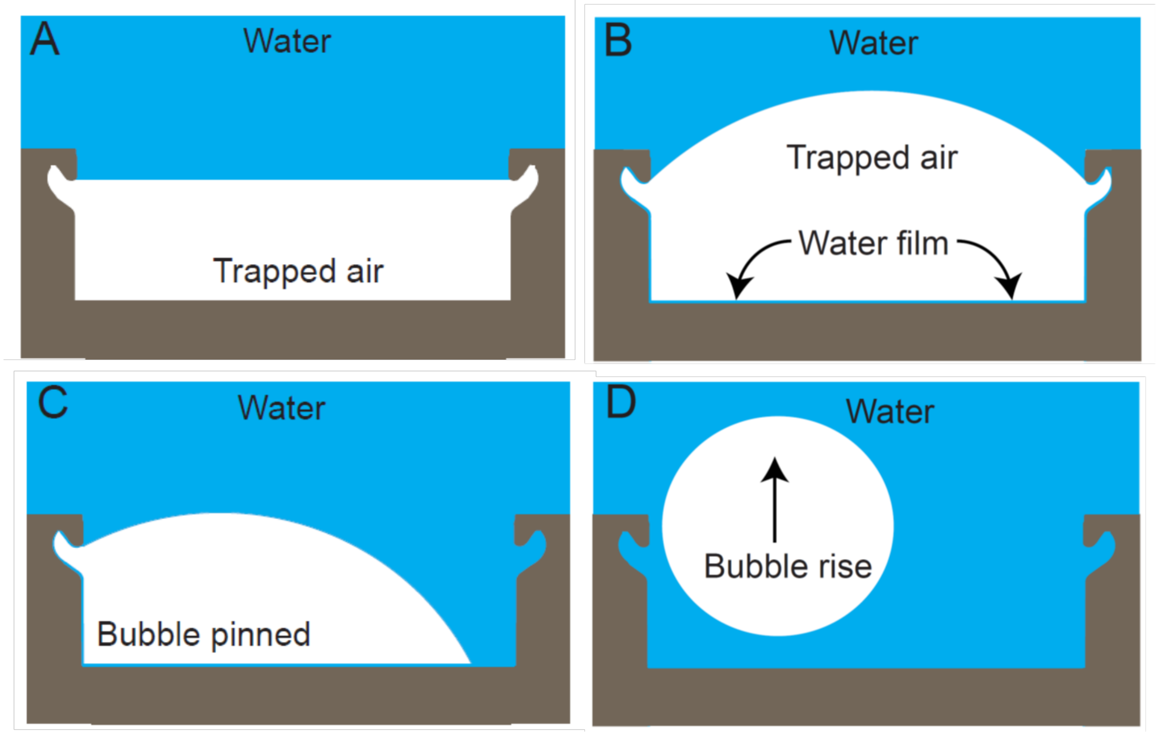

Supplementary Figure 13. **Schematic representation of bubble rise in microtextured silica surfaces treated with oxygen - plasma. (A)** Liquid meniscus is stabilized at doubly reentrant edge. **(B)** Formation of a thin condensed film of water on the cavity wall due to capillary condensation. **(C)** Water enters inside the cavity and meniscus failed asymmetrically. **(D)** The trapped air bubble rises upward due to buoyance and escapes the cavity due to negligible pinning force.

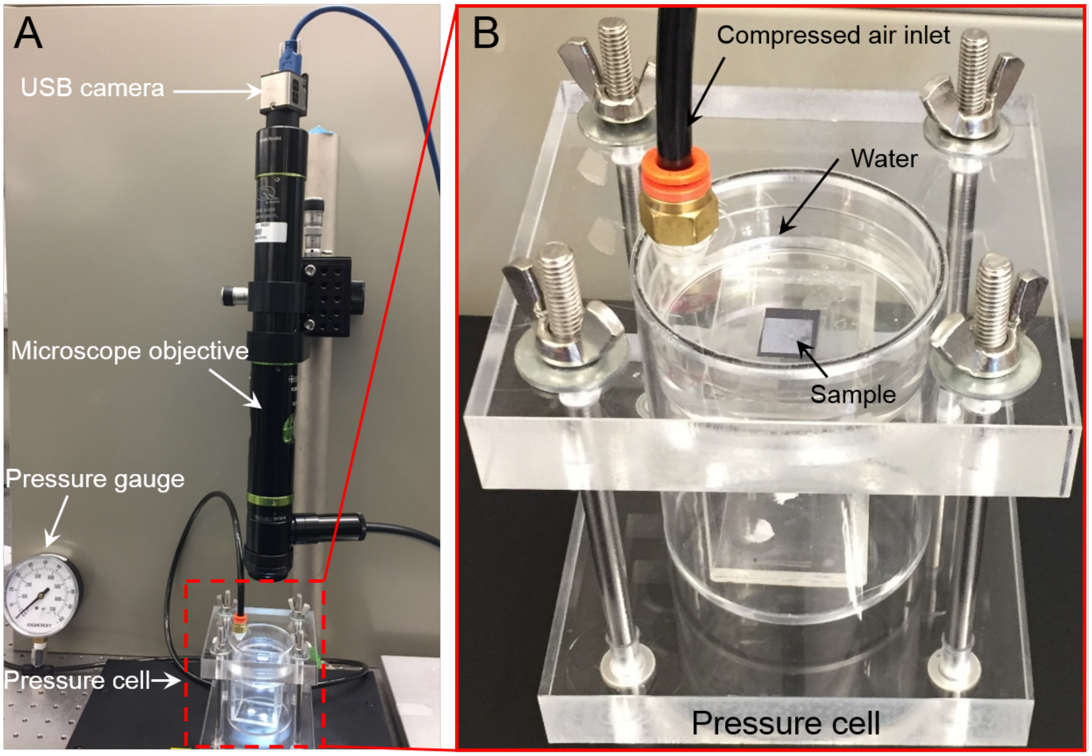

Supplementary Figure 14. **Experimental set-up for measuring breakthrough pressures of cavities**. **(A)** Optical images were recorded using a vertically mounted USB camera. **(B)** Samples were immersed in water inside the pressure cell made of polymethylmethacrylate. Subsequently, we used compressed air to apply external pressure controlled by a regulator.

Supplementary Note 7: Calculation of Breakthrough Pressure

In this section we calculate the breakthrough pressure using ideal gas equation. Supplementary Figure 15 shows the liquid meniscus shapes under applied external pressure using home built pressure cell (Supplementary Figure 14). Since our cavity depth, $h = D/4$, the breakthrough occurred main meniscus touching the cavity floor.

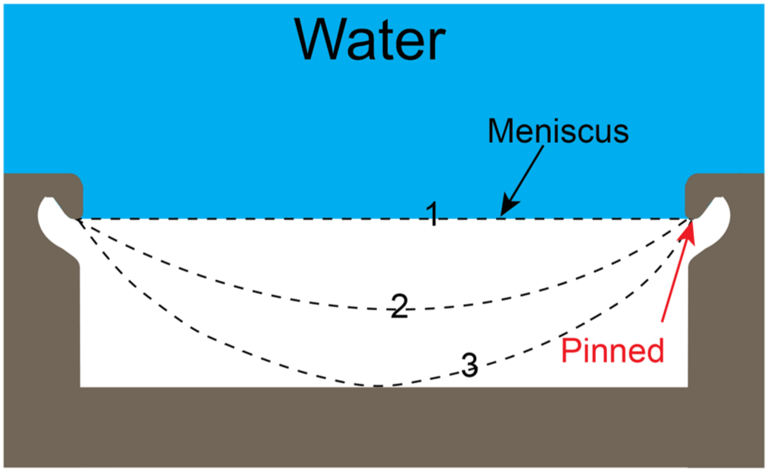

Supplementary Figure 15. Wetting transitions under external pressure. Schematic representation of wetting transitions under external pressures onto microtextured silica surfaces comprising of arrays of doubly reentrant cavities (1) The liquid meniscus is stabilized at the doubly reentrant edge; (2) Application of external pressure pushes the meniscus downwards, still pinned at the doubly reentrant edge, such that it acquires concave curvature. (3) Further increase in external pressure leads to contact between the liquid and the walls of the cavities, where the concave curvature of the liquid-vapor interface touches the cavity floor and liquid inwards.

As the liquid is pushed inward by external force, the pressure inside the cavity rises as $P_2 = P_1V_1/V_2$, where P_1, P_2 and V_1, V_2 are the pressures and volumes of the trapped air right after the immersion and just before the breakthrough(Supplementary Figure 16).
Right after the immersion (Supplementary Figure 16A), pressure inside the cavity, P_1= 1 atm and volume $V_1 = \pi R^2 h$; the geometrical dimensions of the cavity, i.e. depth ($h \approx$ 50 µm), radius (R = 100 µm)

$$V_1 = \pi R^2 h = 1.57 \times 10^6\ (\mu\text{m})^3$$

Under applied external pressure meniscus sagging height (H) increases till it touches the bottom of the cavity wall and fills the entire cavity as shown in Supplementary Figure 16B.

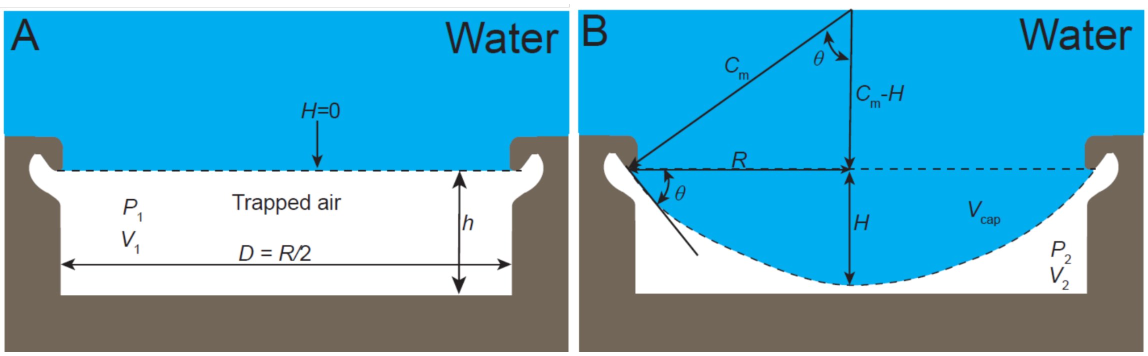

Supplementary Figure 16. Calculation of the volume of the air trapped inside DRCs on immersion in water. Schematic representation of silica surfaces with doubly reentrant cavities: **(A)** right after the immersion – the pressure and volume of the air trapped in the cavity are P_1 = 1 atm and $V_1 = \pi R^2 h = 1.57 \times 10^6$ µm^3 **(B)** In our cavities, as soon as the intruding liquid meniscus touches the bottom of the cavity, breakthrough happens. As the liquid is pushed inward by external force, the pressure inside the cavity rises as $P_2 = P_1 V_1 / V_2$, where P_1, P_2 and V_1, V_2 are the pressures and volumes of the trapped air right after the immersion (1 atm) and just before the breakthrough.

Based on Supplementary figure 16B, mean curvature of the intruding liquid meniscus can be written

$$C_\mathrm{m} = \frac{R^2 + H^2}{2H}$$

And the volume of sagging liquid (V_cap)

$$V_\mathrm{cap} = \frac{\pi H^2 (3C_\mathrm{m} - H)}{3}$$

We used ideal gas equation to estimate the excess pressure (P_b= 116 kPa) inside the cavity at which breakthrough occurs; the geometrical dimensions of the cavity, i.e. depth ($h \approx 50$ µm), radius ($R = 100$ µm) and mean curvature of the intruding liquid meniscus ($C_\mathrm{m} = 125$ µm). The volume of liquid that sags inside the cavity, $V_\mathrm{cap} = 8.4 \times 10^5$ (µm)^3. Final trapped air volume at which breakthrough occurs $V_2 = V_1 - V_\mathrm{cap} = 7.2 \times 10^5$ (µm)^3.

Supplementary Table 2. **Predicted breakthrough pressures**. Table presents the air pressure inside the cavity for the different meniscus sagging height under applied external pressure using ideal gas equation.

Radius of the cavity, *R,* (μm)	Meniscus Sagging height, *H,* (μm)	Mean curvature of the meniscus, C_m, (μm)	Volume of liquid in the cap, V_{cap}, (μm)^3	Volume of trapped air, (μm)^3	Predicted breakthrough pressure, P_b, kPa
100	10	505	156047	1414953	11
100	20	260	315205	1255795	25
100	30	182	480585	1090415	44
100	40	145	655296	915704	72
100	50	125	842449	728551	116

Supplementary References:

1 Butt, H.-J. & Kappl, M. *Surface and Interfacial Forces*. (Wiley-VCH Verlag GmbH & Co., 2010).

2 Lide, D. R. *CRC Handbook of Chemistry and Physics*. Vol. 79 (CRC Press, 1999).

3 Tripathi, N. Densities, viscosities, and refractive indices of mixtures of hexane with cyclohexane, decane, hexadecane, and squalane at 298.15 K. *Int. J. Thermophys.* **26**, 693-703 (2005).

4 Mathuni, T., Kim, J. I. & Park, S. J. Phase equilibrium and physical properties for the purification of propylene carbonate (PC) and gamma-butyrolactone (GBL). *J. Chem. Eng. Data.* **56**, 89-96 (2011).

5 Hesse, P. J., Battino, R., Scharlin, P. & Wilhelm, E. Solubility of gases in liquids .20. Solubility of He, Ne, Ar, Kr, N-2, O-2, CH4, CF4, and SF6 in n-alkanes n-ClH2l+2 (6<=l<=16) at 298.15 K. *J. Chem. Eng. Data.* **41**, 195-201 (1996).

6 Yamamoto, H. & Tokunaga, J. Solubilities of nitrogen and oxygen in 1,2-ethanediol plus water at 298.15-k and 101.33-kpa. *J. Chem. Eng. Data.* **39**, 544-547 (1994).

7 Cassie, A. B. D. & Baxter, S. Wettability of porous surfaces. *T. Faraday. Soc.* **40**, 0546-0550 (1944).

8 Kaufman, Y. *et al.* Simple-to-apply wetting model to predict thermodynamically stable and metastable contact angles on textured/rough/patterned surfaces. *J. Phys. Chem. C.* **121**, 5642-5656 (2017).

9 Mori, Y. H., Vandeven, T. G. M. & Mason, S. G. Resistance to spreading of liquids by sharp edged microsteps. *Colloids. Surf.* **4**, 1-15 (1982).

10 Oliver, J. F., Huh, C. & Mason, S. G. Resistance to spreading of liquids by sharp edges. *J. Colloid Interf Sci.* **59**, 568-581 (1977).

11 Mishra, H. *et al.* Time-dependent wetting behavior of PDMS surfaces with bioinspired, hierarchical structures. *Acs Appl. Mater. Interfaces.* **8**, 8168-8174 (2016).

12 Leger, L. & Joanny, J. Liquid spreading. *Rep. Prog. Phys.* **55**, 431 (1992).

13 Bonn, D., Eggers, J., Indekeu, J., Meunier, J. & Rolley, E. Wetting and spreading. *Rev. Mod. Phys.* **81**, 739-805 (2009).

14 Kulkarni, A. A. & Joshi, J. B. Bubble formation and bubble rise velocity in gas-liquid systems: A review. *Ind. Eng. Chem. Res.* **44**, 5873-5931 (2005).

15 Beysens, D. Dew nucleation and growth. *C. R. Phys.* **7**, 1082-1100 (2006).
