# Supplementary material for: Biomimetic coating-free surfaces for long-term entrapment of air under wetting liquids
Source: Nat Commun. 2018 Sep 6;9:3606. doi: 10.1038/s41467-018-05895-x (PMC6127334; doi:10.1038/s41467-018-05895-x)
Supplement: Supplementary file 2 — Description of Additional Supplementary Files [file 41467_2018_5895_MOESM2_ESM.pdf]

Description of Additional Supplementary Files

File Name: Supplementary Movie 1
Description: Wetting transitions on microtextured silica surfaces with Circ|SC|100 cavities under hexadecane ($\theta_o \approx$ 20°). (Scale: Cavity diameter is 200 μm)

File Name: Supplementary Movie 2
Description: Wetting transitions on microtextured silica surfaces with Sq|SC|3 cavities under hexadecane ($\theta_o \approx$ 20°). (Scale: Cavity edge length is 200 μm)

File Name: Supplementary Movie 3
Description: Wetting transitions on microtextured silica surfaces with Sq|SC|50 cavities under hexadecane ($\theta_o \approx$ 20°). (Scale: Cavity edge length is 200 μm)

File Name: Supplementary Movie 4
Description: Wetting transitions on microtextured silica surfaces with Hex|SC|3 cavities under hexadecane ($\theta_o \approx$ 20°). (Scale: Cavity length is 200 μm)

File Name: Supplementary Movie 5
Description: Wetting transitions on microtextured silica surfaces with Hex|SC|50 cavities under hexadecane ($\theta_o \approx$ 20°). (Scale: Cavity length is 200 μm)

File Name: Supplementary Movie 6
Description: Wetting transitions on microtextured silica surfaces with Circ|SC|100 cavities underwater ($\theta_o \approx$ 40°). (Scale: Cavity diameter is 200 μm)

File Name: Supplementary Movie 7
Description: Wetting transitions on plasma treated microtextured silica surfaces with Circ|DRC|100 cavities in underwater ($\theta_o \approx$ 0°). Meniscus is stabilized at doubly reentrant edge (~1s), but rapid formation of capillary condensed water inside the cavity wall triggers main water meniscus to enter inside the cavity and the trapped air bubble experiences less pinning force and leaves the cavity due to buoyancy. (Scale: Cavity diameter is 200 μm)
